# Defining the robust behaviour of the plant clock gene circuit with absolute RNA timeseries and open infrastructure

**DOI:** 10.1098/rsob.150042

**Published:** 2015-10-14

**Authors:** Anna Flis, Aurora Piñas Fernández, Tomasz Zielinski, Virginie Mengin, Ronan Sulpice, Kevin Stratford, Alastair Hume, Alexandra Pokhilko, Megan M. Southern, Daniel D. Seaton, Harriet G. McWatters, Mark Stitt, Karen J. Halliday, Andrew J. Millar

**Affiliations:** 1 Max Planck Institute of Molecular Plant Physiology, Am Muehlenberg 1, 14476 Potsdam-Golm, Germany; 2 SynthSys and School of Biological Sciences, University of Edinburgh, C.H. Waddington Building, Edinburgh EH9 3JD, UK; 3 EPCC, University of Edinburgh, James Clerk Maxwell Building, Edinburgh EH9 3JZ, UK; 4 Institute of Molecular Cell and Systems Biology, University of Glasgow, Bower Building, Glasgow G12 8QQ, UK; 5 Department of Biological Sciences, University of Warwick, Coventry CV4 7AL, UK

**Keywords:** circadian rhythms, plant biology, gene regulatory networks, biological clocks, model optimization, data management

## Abstract

Our understanding of the complex, transcriptional feedback loops in the circadian clock mechanism has depended upon quantitative, timeseries data from disparate sources. We measure clock gene RNA profiles in *Arabidopsis thaliana* seedlings, grown with or without exogenous sucrose, or in soil-grown plants and in wild-type and mutant backgrounds. The RNA profiles were strikingly robust across the experimental conditions, so current mathematical models are likely to be broadly applicable in leaf tissue. In addition to providing reference data, unexpected behaviours included co-expression of *PRR9* and *ELF4*, and regulation of *PRR5* by *GI*. Absolute RNA quantification revealed low levels of *PRR9* transcripts (peak approx. 50 copies cell^−1^) compared with other clock genes, and threefold higher levels of *LHY* RNA (more than 1500 copies cell^−1^) than of its close relative *CCA1*. The data are disseminated from BioDare, an online repository for focused timeseries data, which is expected to benefit mechanistic modelling. One data subset successfully constrained clock gene expression in a complex model, using publicly available software on parallel computers, without expert tuning or programming. We outline the empirical and mathematical justification for data aggregation in understanding highly interconnected, dynamic networks such as the clock, and the observed design constraints on the resources required to make this approach widely accessible.

## 1. Introduction

Circadian clocks are found widely among organisms from archaea to mammals [1,2]. These internal time-keepers generate approximately 24 h rhythms in the expression of 10–30% of genes, even without environmental cues. In natural conditions, circadian rhythms are entrained by light and temperature cycles. Their function is to coordinate internal processes with the external day/night cycle [3,4] and also, through photoperiodism, relative to the seasonal cycle [5]. The circadian system of each organism includes a phylum-specific gene regulatory network that is required for most rhythmicity [6], as well as non-transcriptional oscillator(s) that are less well characterized in eukaryotes [7].

**Figure 1.**
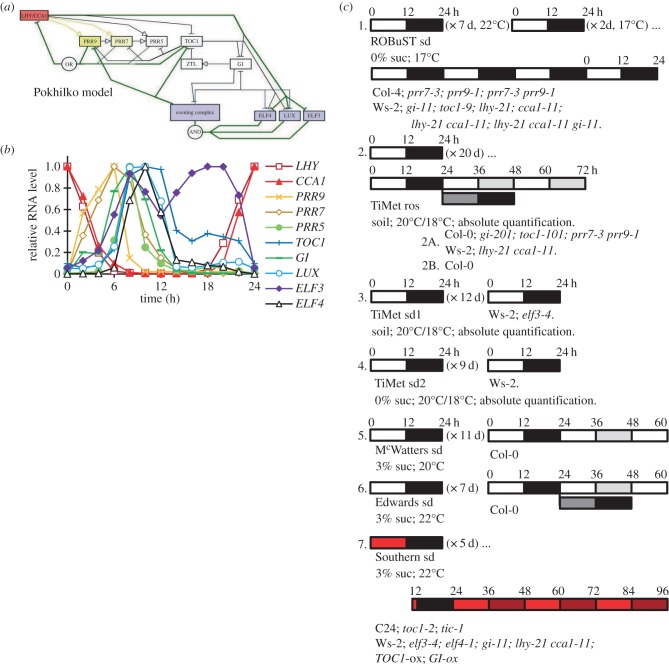
The clock gene network and experimental protocols. (*a*) The clock gene network summarized in the activity-flow language of SBGN v. 1.0 [8], with the principal connections in the P2012 model [9]. The repressilator is denoted by green lines; morning loop components are filled yellow; *LHY/CCA1*, red; evening loop components, blue. Light inputs are shown in electronic supplementary material, figure S1 and all modelled connections of P2011 [10] in electronic supplementary material, figure S2. (*b*) Peak-normalized RNA profiles of genes depicted in (*a*), in plants of the Col-0 accession under a 12 h light : 12 h dark cycle (LD 12 : 12; experiment 2*b* of panel (*c*)). (*c*) Graphical representation of the growth conditions. Experiments 1, 4, 5, 6 and 7 used seedlings grown in LD 12 : 12 for the number of days indicated; experiments 2 and 3 used plants grown on soil in LD 12 : 12 for the number of days indicated. Sucrose concentrations, growth temperatures and genotypes tested are shown for each experiment. Open box, light interval; black box, dark interval; light grey box, predicted darkness in constant light; dark grey box, predicted light in constant darkness; red box, red light. Sampling time in ZT (h), relative to lights-on of the first day of sampling or the last dawn before experimental treatment (ZT0). Ros, rosette; sd, seedling.

In plants, the clock gene network includes highly connected, negative regulators forming a complicated circuit. This has been best studied in *Arabidopsis thaliana*. One simplification (figure 1*a*) visualizes the circuit as a three-loop structure of morning and evening loops coupled around a repressilator [10,11]. The morning loop includes the MYB-related transcription factors LHY and CCA1, which activate expression of the pseudo-response regulators *PRR9* and *PRR7* [12,13], but inhibit expression of later-expressed genes including *PRR5* and *TOC1* (*PRR1*). PRR9, PRR7, PRR5 and TOC1 bind to and inhibit *LHY* and *CCA1* expression, as predicted by modelling [10,14] and demonstrated by experiments [15–18]. LHY and CCA1 also inhibit expression of *ELF3, ELF4* and *LUX* (*PCL1*), whose protein products interact to form another repressor, the evening complex (EC) [19–22]. The EC is thought to inhibit the expression of at least *ELF4* and *LUX*, forming a negative feedback loop, whose continued function might explain the damped oscillation of clock gene expression observed in *lhy cca1* double mutant plants [10]. GI, a large plant-specific protein, is also rhythmically expressed but functions at a post-translational level through, for example, stabilization of the TOC1-degradation factor ZTL [23]. Light signalling controls multiple processes (electronic supplementary material, figure S1) that entrain the clock circuit to the day–night cycle. A growing number of identified processes and components remain to be fully integrated into the circuit, though even the components described are challenging to analyse.

Formal, mathematical models have been helpful in understanding the plant clock, because its components are highly interconnected by nonlinear regulation (electronic supplementary material, figure S2; reviewed in [24]). Model development was necessarily based upon timeseries data, where the system was manipulated using mutations and by varying light or temperature inputs. More detailed models demanded greater precision and breadth in the data, which raised two major issues. First, data collation was laborious, because the numerical data underlying published timeseries graphs were rarely accessible [25]. Although the potential benefits of data sharing are recognized [26,27], in practice, useful sharing requires cyber infrastructure, which is currently best-developed for omics data rather than the many focused studies in the clock literature [28]. Second, the published data on *Arabidopsis* clocks used several genetic backgrounds and growth conditions, introducing ill-defined variation to the results.

To provide directly comparable data, we conducted large-scale qRT-PCR assays for the RNA levels of multiple clock genes. Overlapping studies in four laboratories using different growth stages and conditions highlighted the robustness of most expression profiles and the few instances where they varied. Visualizing the data as phase plane plots suggested new dynamic interactions and their genetic regulators. Absolute RNA quantification revealed the low expression levels of *ELF3* and *PRR9*. To facilitate similar projects, we introduce data aggregation in the online BioDare resource, and illustrate the utility of our datasets by reoptimizing the P2011 clock model [10] with the open-source application Systems Biology Software Infrastructure (SBSI) [29], highlighting key areas for future experiments.

## 2. Results

### 2.1. Large-scale measurement of clock gene RNA profiles

This study was motivated by two projects that integrated circadian regulation into research on other plant physiological systems, which were incompatible with the growth conditions used in earlier circadian research. The Regulation of Biological Signalling by Temperature (ROBuST) project studied the interactions of ambient temperature with circadian and light signalling circuits; exogenous sucrose inhibits light signalling [30,31] and was therefore excluded. The Timing of Metabolism (TiMet) project studied circadian regulation of the starch pathway, among others, which is best characterized in rosette plants grown on soil. To measure the rhythmic expression in a set of clock-related genes (figure 1*b*), we used automated systems in Golm and Edinburgh to quantify mRNA levels for components of the clock circuit every 2 h, in multiple conditions and mutant backgrounds [32,33] (figure 1*c*). The ROBuST dataset tested 13-day-old, wild-type (WT) and mutant seedlings grown at 17°C on agar medium without additional sucrose. Datasets from the TiMet project tested 21-day-old rosette plants grown at 20°C on soil (TiMet ros) and 13-day-old seedlings on soil (TiMet sd1). The TiMet rosette data were collected from WT and clock mutant *Arabidopsis thaliana* plants grown under light : dark (LD) cycles in two experiments, followed by constant light (LL) or constant dark (DD) in one study. Three further studies were compared, from seedlings grown on sterile agar media without sucrose (TiMet sd2, using the same medium as the ROBuST data), or with exogenous sucrose under white (McWatters, this paper; and Edwards *et al*. [34]) or red light (Southern, this paper; and [21,35]).

### 2.2. Data presentation

**Figure 2.**
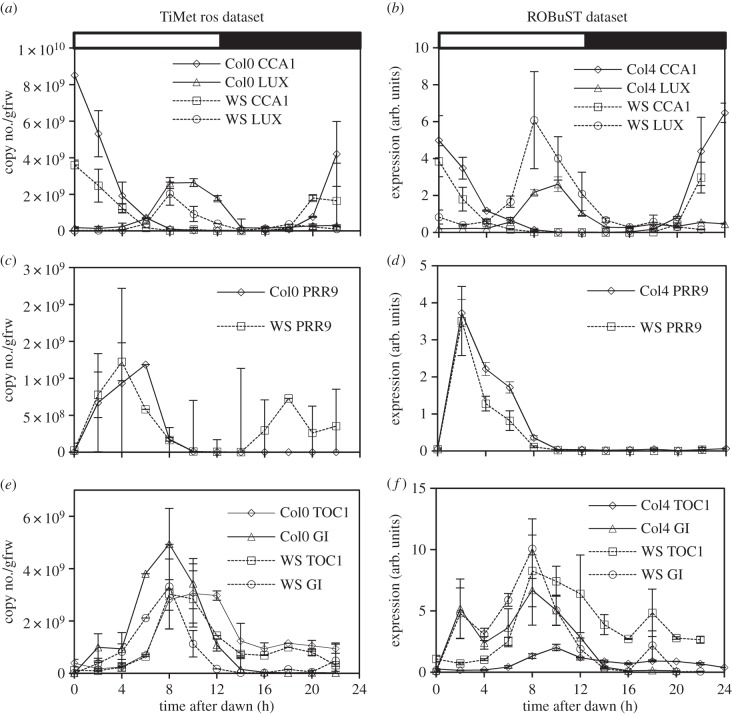
Clock gene expression in wild-type plants under LD cycles. Transcript levels in Col-0 and Ws-2 WT under LD 12 : 12 were measured by qRT-PCR, in experiment 2 (TiMet ros) including eight external RNA standards to allow absolute quantification in Col-0 and Ws-2 (*a,c,e*) and in experiment 1 (ROBuST) normalized to the *ACTIN7* control in Col-4 and Ws-2 (*b,d,f*). Data represent transcripts of (*a,b*) *LHY* and *CCA1*, (*c,d*) *PRR9*, and (*e,f*) *TOC1* and *GI*. Error bars show SD, for two to three biological replicates. Electronic supplementary material, figure S3 shows the data on logarithmic plots.

**Figure 3.**
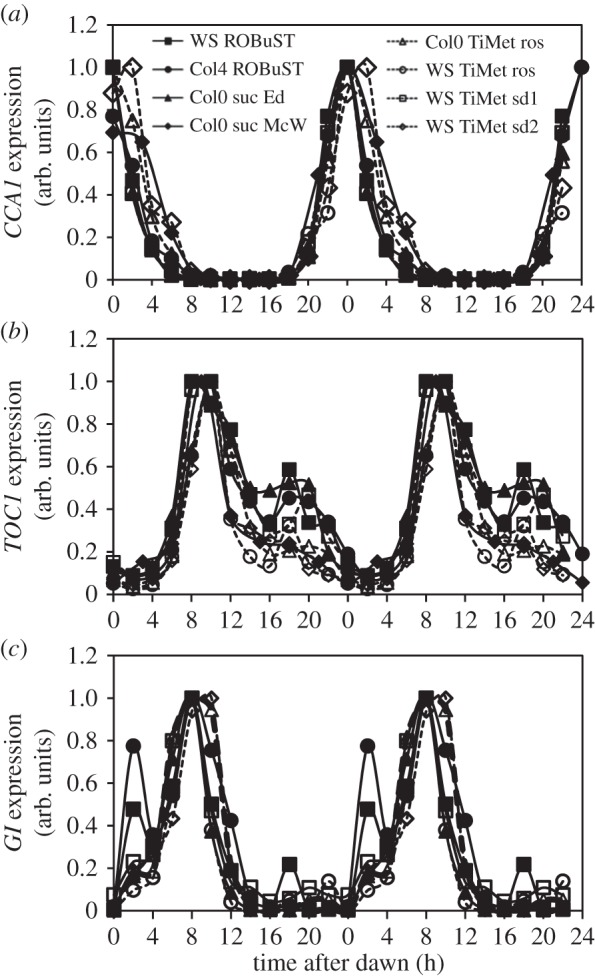
Waveforms of clock gene expression across experiments at different plant age and in the absence and presence of exogenous sucrose. This plot compares transcript abundance of *CCA1*, *TOC1* and *GI* in 12 h photoperiods in three WTs grown in different experimental conditions in different laboratories. The data are taken from the following experiments (figure 1): WS ROBuST (1, seedlings), Col4 ROBuST (1, seedlings), Col0 suc Ed (6, seedlings provided with 3% exogenous sucrose), Col0 suc McW (5, seedlings provided with 3% sucrose), Col0 TiMet ros (2B, 21 day-old rosettes), WS TiMet ros (2, 21 day-old rosettes), WS TiMet sd1 (3, 10 day-old seedlings), WS TiMet sd2 (4, 13-day-old seedlings). All plants were entrained in LD 12 : 12 (figure 1). Values for each transcript are normalized to the peak. The results are the mean of duplicate or triplicate samples, double-plotted; error bars are not shown for clarity.

**Figure 4.**
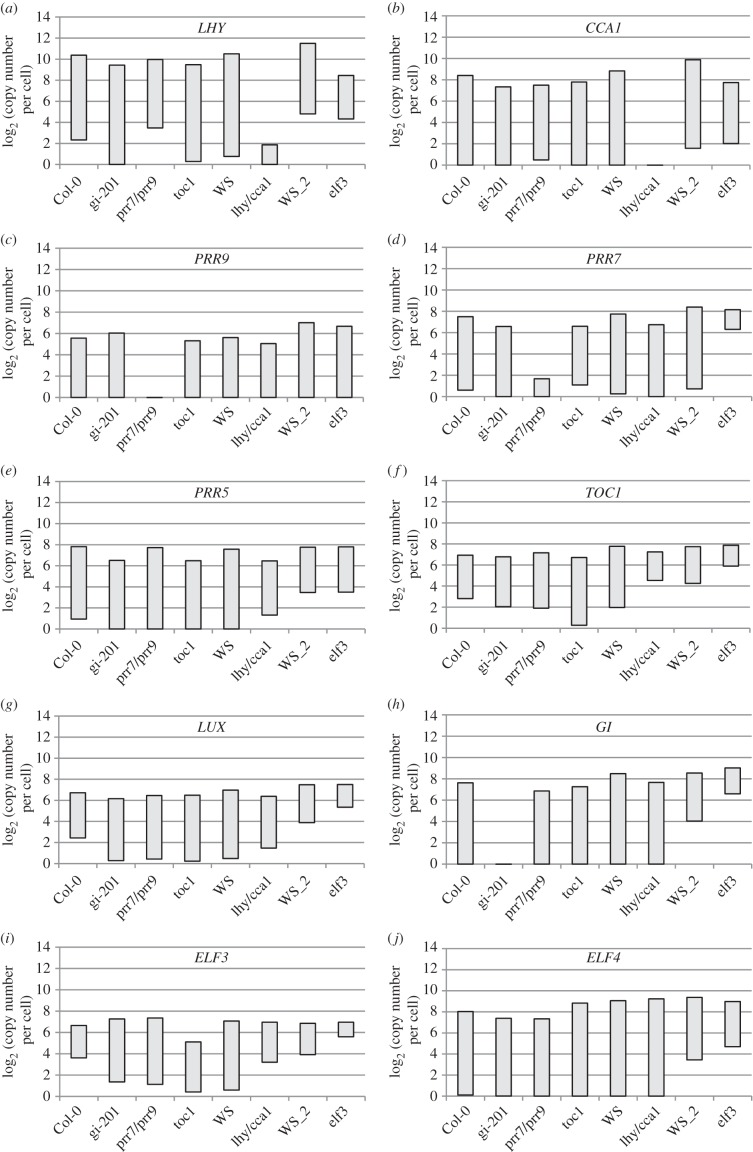
Range of transcript abundance for clock genes in clock mutants. The bars show the highest and lowest mean values for the absolute abundance of transcripts for clock genes in a given genotype. The genotypes are, from left to right, Col-0 wild-type, *gi-201*, *prr9 prr7* double mutant, *toc1*, WS WT, *lhy cca1* double mutant (from experiments 2 and 2B of figure 1*c*, 21-day-old rosettes) and WS (designated WS_2) and *elf3* from experiment 3 (13-day-old seedlings), (*a*) *LHY*, (*b*) *CCA1*, (*c*) *PRR9*, (*d*) *PRR7*, (*e*), *PRR5*, (*f*), *TOC1*, (*g*) *LUX*, (*h*) *GI*, (*i*) *ELF3*, (*j*) *ELF4*. The underlying data are as in figures 5 and 6.

**Figure 5.**
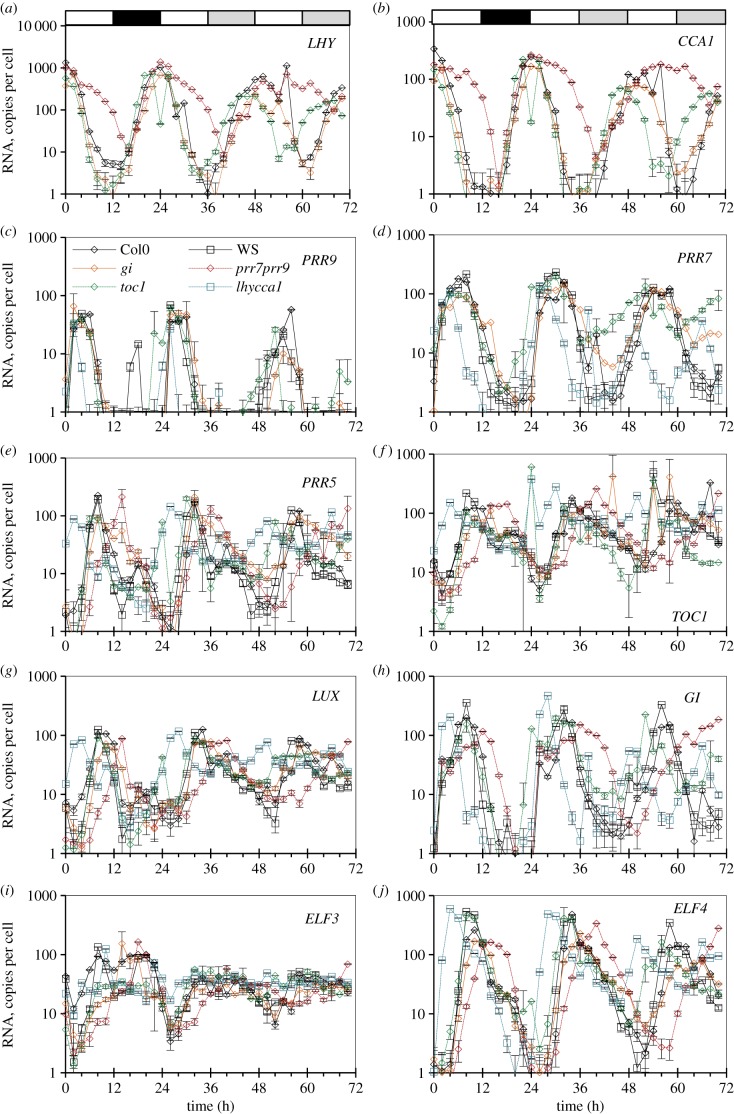

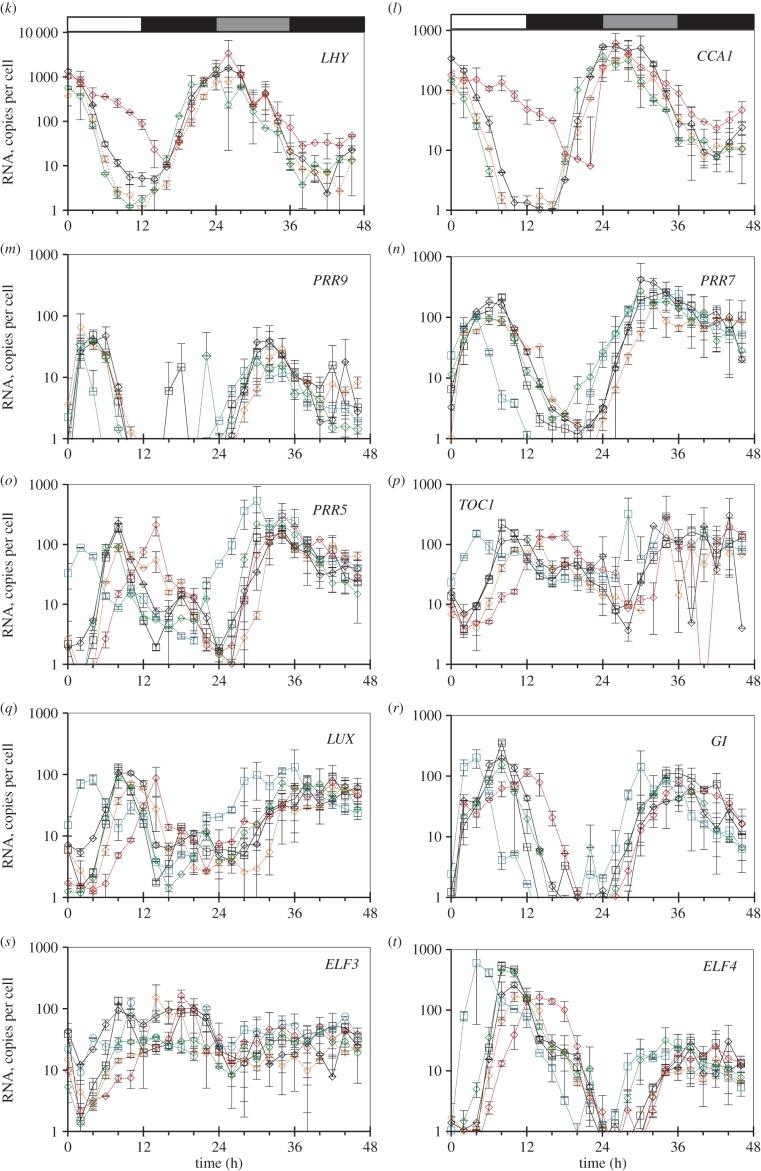
Clock gene expression in wild-type plants and clock mutants in LD, and after transition to constant light (LL) or darkness (DD). Col-0 and Ws-2 WT, the *lhy-21 cca1-11* and *prr7-3 prr9-1* double mutants, and the *toc1-101* and *gi-201* single mutants were grown in a 12 h photoperiod for 20 days, harvested through a LD cycle and then transferred to LL (*a–j*) or DD (*k–t*; TiMet ros, dataset 2 of figure 1*c*). Transcript levels for clock genes were measured by qRT-PCR, including eight external RNA standards to allow absolute quantification. (*a,k*) *LHY*, (*b,l*) *CCA1*, (*c,m*) *PRR9*, (*d,n*) *PRR7*, (*e,o*), *PRR5*, (*f,p*), *TOC1*, (*g,q*) *LUX*, (*h,r*) *GI*, (*i,s*) *ELF3*, (*j,t*) *ELF4*. The results are the mean of duplicate samples, error bars show SD. Open box, light interval; black box, dark interval; light grey box, predicted darkness in LL; dark grey box, predicted light in DD.

**Figure 6.**
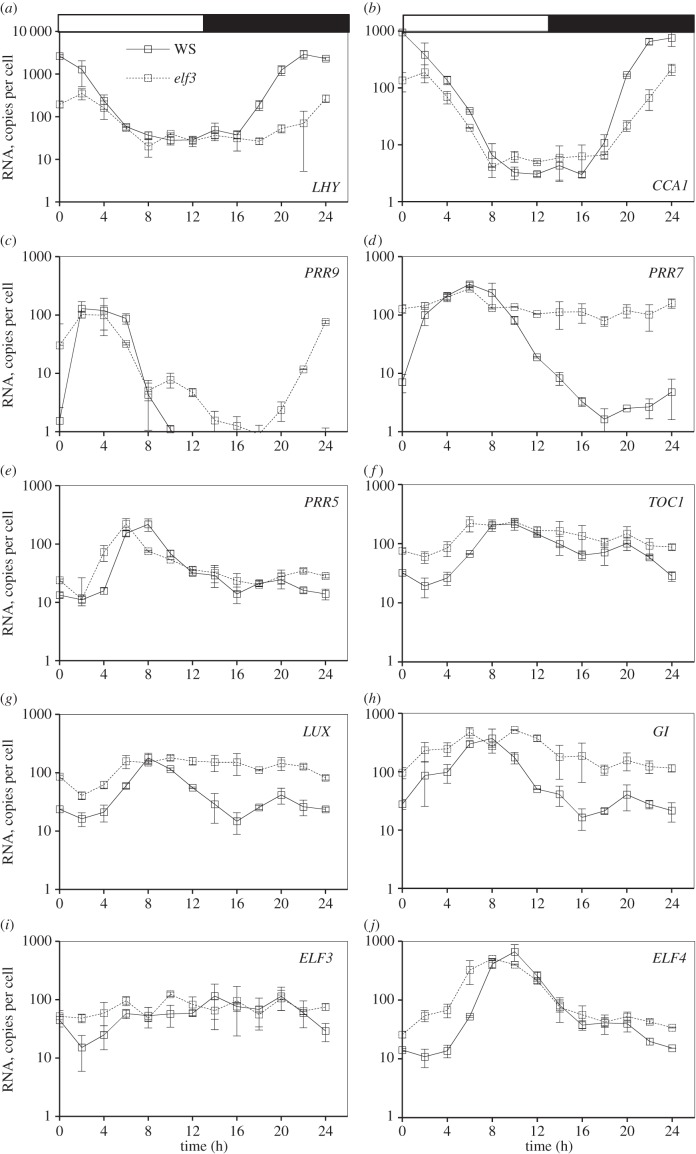
Clock gene expression in wild-type plants and *elf3* mutants in LD. Ws-2 WT (solid lines) and *elf3–4* mutant plants (dashed lines) were grown in a 12 h photoperiod for 12 days and harvested through one LD cycle (TiMet sd, dataset 3 of figure 1*c*). Transcript levels for clock genes were measured by qRT-PCR, including eight external RNA standards to allow absolute quantification. (*a*) *LHY*, (*b*) *CCA1*, (*c*) *PRR9*, (*d*) *PRR7*, (*e*), *PRR5*, (*f*), *TOC1*, (*g*) *LUX*, (*h*) *GI*, (*i*) *ELF3*, (*j*) *ELF4*. The results are the mean of duplicate samples. Error bars show SD.

Time is expressed as zeitgeber time (ZT) in hours since the last dark–light transition, by convention; the first dark–light transition within the sampling interval is 0 h on our plots. TiMet data are presented as absolute values [33], obtained by calibrating RNA extraction efficiency with heterologous control RNAs (electronic supplementary matetial, table S1) to calculate the number of copies of each RNA per gram fresh weight (gFW). Estimated cell numbers per gFW (see electronic supplementary material) were used to calculate RNA copies per cell. The other datasets are normalized relative to a control transcript (*ACTIN7* for ROBuST; *ACTIN2* for Edwards and Southern; *βTUBULIN4* for McWatters). *ACTIN2* and *GAPDH* controls were also assayed with two amplicons each in the TiMet assays, for comparison among datasets. Data were replicated in biological duplicate or triplicate samples and in equivalent sampling on successive days (0–12 h and 24–36 h in the TiMet and Edwards datasets). Data are presented on linear scales to reflect the potential for protein synthesis and hence regulatory effects on downstream targets (in keeping with most of the literature; figures 2 and 3; electronic supplementary material, figure S5) and on logarithmic scales to reveal the full dynamic range of RNA expression, and hence the influence of multiple upstream regulators (figures 4–6; electronic supplementary material, figure S3 and S4). Further technical comparison among the studies is presented in the electronic supplementary material.

### 2.3. Similarity and specific variations of wild-type RNA profiles across datasets

Clock gene RNA expression profiles in WT plants of two accessions (Col and Ws-2) grown in LD are presented in figure 2; profiles were similar across the TiMet and ROBuST datasets, despite major differences in growth conditions. The morning clock components, *CCA1* and *LHY*, peaked as expected at dawn (figure 2*a,b*), followed by *PRR7* (ZT6; figure 2*c,d*), *PRR5* and *GI* (ZT8; figure 2*e–h*). Expression of the evening components, *LUX, ELF4* and *TOC1*, peaked at ZT8–12 (figure 2*e–j*); peak expression of *LUX* was delayed by about 2 h in Col plants relative to Ws-2 in both datasets (figure 2*g,h*; replicated in LL data). *ELF3* had a low-amplitude profile in both datasets, with lowest expression around ZT4.

The TiMet and ROBuST datasets differed at particular timepoints for *PRR9, GI* and *TOC1*. *PRR9* expression was highest at ZT2–6 in both cases, with a clear peak at ZT2 in the ROBuST seedling data (consistent with many other reports from seedlings) but a broader profile in the TiMet data (figure 2*c,d*). After its major peak at ZT8–12, *TOC1* expression is consistently observed (since [36]) to increase around ZT18, but the level of this night-time peak varied (figure 2*e,f*). The ROBuST data for seedlings showed a peak of *GI* expression at ZT2 (figure 2*f*); little induction is evident at ZT2 in the TiMet rosette data on a linear scale (figure 2*e*) though the logarithmic scale reveals the response (electronic supplementary material, figure S3*e*). The morning peak in *GI* is likely to be an acute response to lights-on. Rapid sampling in the Southern data [35] and in a follow-up microarray study [10] suggested that induction is rapid but transient, and therefore sensitive to sampling time. Nonetheless, the data suggest that either the magnitude or kinetics of light responsiveness vary across the conditions tested. The difference in *PRR9* profiles could reflect slower activation of *PRR9* in the TiMet data, consistent with lower light responsiveness in rosettes than in seedlings or with faster repression of *PRR9* in seedlings. The level of *GI* transcripts at ZT12 also varied from 4% to 40% of the peak level, with the lowest level in rosettes of Ws-2 (figures 2*e,f* and 3*c*). *GI* expression is light sensitive at this phase [37], so our results are consistent with variation in light responsiveness.

Sucrose modestly increases expression of the evening clock components *TOC1* and *GI* [38], particularly in darkness [39], and can repress *PRR7* with subsequent effects on *CCA1* under low light [40], along with transcriptome-wide effects under LD cycles [41,42]. We therefore compared the expression profiles for *CCA1, TOC1* and *GI* in plants grown without (ROBuST and TiMet data) or with exogenous sucrose (McWatters, Edwards and Southern datasets; figure 3). To facilitate comparison, TiMet data were normalized to control transcripts (two amplicons each in *GAPDH* and *ACTIN2*), as for the other studies. Each profile was normalized to its maximum. Expression profiles of *CCA1* across the different timeseries matched closely despite the differences in accession and experimental protocols (figure 3*a*). The times of peak, mid-rising and mid-falling phases differed by at most 2 h (one sampling interval) among datasets. In the falling phase at ZT4, the profiles in McWatters, TiMet ros and TiMet sd2 data were delayed relative to the other data. The night-time expression of *TOC1* at ZT18 varied from 20% to 60% of the main peak level (figure 3*b*), with high expression in ROBuST, Edwards and TiMet sd2 datasets. The expression of *GI* at ZT2 in the TiMet and Edwards seedling data was about 20% of the main peak level (figure 3*c*, also in Southern data [35]), intermediate between the levels in ROBuST and TiMet rosette data (discussed above). These features of the expression profiles showed no clear relationship with growth medium or developmental stage.

### 2.4. Absolute quantification of clock gene transcripts

The absolute quantification in the TiMet ros data, which is based ultimately upon the certified amounts of synthetic commercial standards [33], revealed wide variation in peak RNA levels among clock genes in WT plants (figure 4). Highest RNA levels were detected for *LHY* at 1000–2100 copies per cell, similar to the control genes *GAPDH* and *ACT2*. *PRR9* was least abundant at the peak, with 40–70 copies per cell; *LUX* and *ELF3* peaked at 105–130 copies per cell; *PRR7, PRR5, GI* and *TOC1* at 120–270 copies per cell; *ELF4* and *CCA1* at 250–600 copies per cell. RNA copy number of *LHY* was threefold greater than that of *CCA1* (figure 4*a,b*).

Peak levels for the evening-expressed genes (figure 4*f–j*) were slightly higher in Ws-2 than Col-0 plants, by 1.2-fold (*LUX*) to 2.0-fold (*ELF4*), average 1.6-fold. Several clock gene RNAs fell to low copy numbers per cell at the trough. Consequently, rhythmic amplitudes (defined here as peak divided by trough levels) also varied greatly among clock genes. The *TOC1* and *ELF3* profiles showed only eight- to 20-fold amplitude in Col-0, and generally smaller amplitudes in other, mutant genotypes than the other clock genes (figure 4*f,i*), whereas *LHY*, *CCA1*, *GI, ELF4* and *PRR5* RNAs showed over 100-fold amplitude. This distinction was consistent in other datasets [21,34]. Amplitude estimates can be significantly affected by variation in the very low trough levels, which were higher in the TiMet sd1 dataset relative to the TiMet rosette data for *LHY* and all the evening-expressed genes in the Ws-2 accession, for example (figure 4). Transcripts with high-amplitude profiles might be expected to control circadian timing more effectively than the low-amplitude profiles of *TOC1* and *ELF3*.

### 2.5. Regulation of clock genes under environmental and genetic manipulation

The TiMet project measured clock gene expression in LL and DD following LD entrainment, in seedlings of two WT and four clock mutant backgrounds (figure 5), revealing novel aspects of clock gene regulation as well as replicating regulation observed in many earlier, smaller studies. The results are discussed below with respect to the upstream regulators of each gene, rather than the effect on the gene's downstream targets. The RNA data are therefore presented in semi-logarithmic plots that show regulator activity even at low RNA levels.

Comparing the three environmental conditions, peak RNA expression levels tended to fall in LL, consistent with the loss of dark-dependent regulation. The acute gene induction at the dark–light transition, faster degradation of PRR repressors in darkness and of the EC in the light are all expected to enhance rhythmic amplitude in LD. Expression levels of the clock RNAs were maintained in the first cycle in DD, except for the strongly light-regulated *ELF4* [43,44]. Comparing the six genotypes, mutations that removed the repressors revealed the key connections in the clock circuit (figure 1*a*). The *gi* mutation, in contrast, had small or negligible effects on the timing and levels of expression except for *PRR5*, as noted below.

#### 2.5.1. *LHY* and *CCA1*

Our results are consistent with PRR repressors controlling both the rising and falling phases of *LHY* and *CCA1* expression at the transcriptional level [14,16–18,45]; several observations suggest that this activity is light-dependent. Both transcripts retain strikingly higher expression in the *prr7;prr9* double mutant than in the WT, at ZT6–12 in LD and LL (figure 5*a,b*; *p* < 0.05; 20- to 30-fold higher at ZT8), consistent with the absence of repression from PRR9 and PRR7 proteins. By the second day in LL, the trough of *LHY* and *CCA1* expression at ZT44 (68 h in figure 5) was also 20-fold higher than the WT trough level at ZT36–38 (60–62 h). Comparing LD and LL data with DD conditions revealed broader peaks of *LHY* and *CCA1* RNA in DD (figure 5*k,l*), consistent with slower degradation of these transcripts in darkness [34,46]. In darkness, however, *LHY* and *CCA1* levels in the *prr7;prr9* mutant behaved very similarly to the WT, both during the falling phase in DD (ZT28–38; figure 5*k,l*) and during the rising phase in LD (ZT16–22; figure 5*a,b*). By dawn in LD, both transcripts peaked at the WT level, consistent with previous reports [12,13]. Thus, the misregulation of *LHY* and *CCA1* in the light in the *prr7;prr9* double mutant was abolished during the dark in LD.

Removing TOC1, the last of the PRR repressors to be expressed, would be expected to allow an earlier rise in expression of *LHY* and *CCA1* during the night in the *toc1* mutant under LD. This effect was relatively small (two- to 2.5-fold higher at ZT18, *p* = 0.02). *LHY* and *CCA1* levels in *toc1* mutants differed less than fourfold from WT at any point in LD. The mutant phenotype was not enhanced in the first DD cycle (figure 5*k,l*). In LL, however, *LHY* and *CCA1* expression in the *toc1* mutant peaked at ZT22 (46 h in figure 5) rather than at ZT26 (50 h) in Col, reached only 30–50% of WT peak level consistent with earlier data [47], and fell much earlier than the WT (19- to 27-fold lower at ZT30, time 54 h in figure 5*a,b*). Thus, the molecular phenotypes of both *prr7;prr9* and *toc1* mutants were light-dependent.

The *elf3* mutant reduced peak expression of both *LHY* and *CCA1* by five- to 10-fold (figure 6*a,b*; electronic supplementary material, figure S5*g*), with greatest effects at ZT20–24. This effect is thought to be indirect, as the EC (comprising ELF4, ELF3 and LUX) is proposed to repress the *PRR*s (figure 1*a*), as well as *LUX* and *ELF4* [10,19,20,22]. De-repression of *PRR* expression in mutants of the EC should therefore explain the effects of *elf3* on *LHY* and *CCA1*.

#### 2.5.2. *PRR9* and *PRR7*

*PRR7* was the most severely affected gene in the *elf3* mutant under LD, maintaining 25–85% of the WT peak level at all times (figure 6*d*), consistent with de-repression of the *PRR7* promoter [21]. The resulting, 30- to 50-fold overexpression of *PRR7* in *elf3* at ZT20–24 is consistent with reduced expression of *LHY* and *CCA1* at this time*. PRR9* transcript levels retained a 100-fold rhythmic amplitude under LD in the *elf3* mutant, indicative of ELF3-independent regulation (see Discussion). Nonetheless, *PRR9* expression was also de-repressed from ZT10 in *elf3* (*p* = 0.05), rising 2–4 h before dawn (figure 6*c*), and presumably also contributing to reduce *LHY* and *CCA1* expression.

The early-expressed *PRRs* are thought to be repressed by the later-expressed *PRR5* and *TOC1* (figure 1*a*). The *toc1* mutation had modest effects on *PRR9* or *PRR7* profiles under LD cycles (figure 5*c,d*), though the changes observed (such as an early rise in *PRR7* at ZT20–24) were not consistently significant in the TiMet and ROBuST datasets, or in DD in the TiMet data (figure 5*n*). *toc1* also had little effect on *LHY* and *CCA1* levels in these conditions (figure 5*a,b*). In LL, however, removing TOC1 prevented full repression of the *PRR*s. The trough of *PRR7* expression was at a 10-fold higher level than in the WT (*p* < 0.05) and 8 h earlier (ZT12 rather than ZT20, 36 h rather than 44 h in figure 5*d*). Higher expression of the repressor *PRR7* at 38–52 h (figure 5*d*) is consistent with the lower peak expression of *CCA1* and *LHY* in *toc1* under LL (figure 1*a* [9]). Taken together, these results suggested that TOC1 repressor function was most effective under constant light conditions, where the *toc1* mutant was originally identified [48].

Light-dependent regulation was also evident in WT plants. Peak *PRR9* expression levels fell less than twofold in the first cycle of DD (*p* > 0.16; figure 5*m*). Peak *PRR7* expression tended to increase (threefold or less) in all genotypes in DD (figure 5*n*; electronic supplementary material, figure S4*c*). The *gi* mutant was an exception, which slowed the rise of all the transcripts in DD except *ELF3* and *ELF4* (figure 5*k–t*; electronic supplementary material, figure S5*d*). Peak expression for some genes was reduced in *gi* below WT levels, including *PRR7* (*p* = 0.02–0.03 at ZT26–28 h). Trough RNA levels in the WT plants rose more dramatically in DD, for *PRR7* and other clock genes (except for *LHY*): the lowest expression of *PRR7* in Col was 1.5 ± 0.4 copies per cell at ZT20 but 65 ± 6.8 copies per cell at ZT40 (electronic supplementary material, figure S4*c*). The Edwards dataset showed similar de-repression of *CCA1* and *GI* trough levels in DD (electronic supplementary material, figure S4*a*,*b* [34]). Lastly, we tested the effect of CCA1 and LHY on the *PRR* transcripts, using the *lhy;cca1* double mutant. In the WT, the repression of the evening-expressed genes by LHY and CCA1 in the early day delays the expression of these and other target genes until the evening. The double mutation advanced the peak phase of all the other clock genes to ZT2–4, as expected, except for *ELF3* (see below). Despite the de-repression, peak levels were not consistently increased relative to the Ws-2 control. Peak expression of *PRR9, PRR7* and *PRR5* (figure 5*c–e*) was slightly reduced (up to twofold) in the *lhy;cca1* background under LD, consistent with earlier results [12]. By ZT8 (or ZT4 for *PRR9*), all the clock genes were expressed at lower levels in *lhy;cca1* than in the WT (*p* < 0.01–0.04), consistent with expression of all the PRR repressors. In the *lhy;cca1* double mutant in DD, however, the *PRR* genes had broad peaks that rose earlier than in the WT (ZT22–30) but did not fall earlier (ZT34–40; figure 5*m–o*; electronic supplementary material, figure S4*d*). The absence of early repression in DD again suggests that inter-regulation of the *PRR*s is light-dependent.

#### 2.5.3. *PRR5* and *TOC1*

The later-expressed *PRR*s are repressed by LHY and CCA1, so longer expression of *LHY* and *CCA1* in the *prr7;prr9* double mutants delayed their expression in LD and LL conditions (figure 5*e,f*), as expected. In contrast, under DD conditions, *PRR5* expression in *prr7;prr9* rose indistinguishably from the WT at ZT26–34 h and peaked slightly (twofold) above the WT level (figure 5*o*). The *lhy;cca1* double mutant caused the phase advance noted above, as the loss of LHY and CCA1 repressors increased *TOC1* levels in the early day. Peak *TOC1* RNA levels in the *lhy;cca1* mutant did not change consistently from WT levels in the TiMet data under LD (figure 5*f*), and were lower than the WT in the ROBuST dataset (*p* < 0.01; electronic supplementary material, figure S5*c*).

Our detailed datasets also allowed us to compare expression waveforms. For example, *PRR5* rises and falls 10-fold within 5 h in both TiMet and ROBuST data (figure 5*e,o*; electronic supplementary material, figures S3*g*,*h*, S5*a*). This narrow peak indicates highly nonlinear control, consistent with negative autoregulation and/or inhibition by TOC1 [15,49]. Moreover, our results indicate that this *PRR5* waveform depends upon GI function. The *gi-201* mutant had limited effects overall but slowed the fall in *PRR5* mRNA in LD and LL (figure 5*e*), creating an asymmetric profile in *PRR5* RNA that was also observed in the *gi-11* tested in the ROBuST data (electronic supplementary material, figure S5*a*,*b*). Repression by the EC might also contribute to the falling phase of the *PRR5* profile. Removing this repression in the *elf3* mutant resulted in moderate de-repression of *PRR5* and *TOC1* in the late night (*p* < 0.01, ZT0/24; *p* = 0.01 for *PRR5* ZT22) and potentially in the early morning (*p* = 0.06–0.08; ZT2–4; figure 6*e,f*). In contrast, de-repression of the early *PRR*s in *elf3* was greatest in the early night (see above), indicating that the profile of regulators varies among the PRR family members (see Discussion).

#### 2.5.4. GI

The main peak of *GI* expression in the late day behaves similarly to *PRR5*, with delayed expression in the *prr7;prr9* double mutant owing to longer expression of LHY and CCA1 under LD and LL but not DD, and an advanced phase in the *lhy;cca1* double mutant (figure 5*h,p*). In contrast to *PRR5* but similarly to *PRR9* and *PRR7, GI* was de-repressed from ZT10 in the *elf3* mutant (*p* < 0.01), consistent with [21] and the Southern dataset (electronic supplementary material, figure S5*f*). The Southern dataset showed that the expression of *GI* was similar in *elf3* and *elf4* mutants, but there was much less effect on *CCA1* in *elf4* than *elf3* (electronic supplementary material, figure S5*g*), indicating that the effects of the EC components can be distinct.

#### 2.5.5. ELF3

The *ELF3* rhythmic profile has low amplitude, as noted above, with a trough at ZT2–4 and peaks at both ZT8 and ZT18–20 in WT plants under LD in the TiMet and ROBuST datasets (figure 5*i,s*; electronic supplementary material, figures S3*i*–*j*, S5*d*). The trough of ELF3 expression is de-repressed at ZT4 in the *lhy;cca1* double mutant (*p* < 0.01), though there is no peak at this time, in contrast to all the other clock genes. The rise in *ELF3* expression is delayed in the *prr7;prr9* double mutant (*p* < 0.01–0.05, at ZT6–10), consistent with repression by increased levels of LHY and CCA1 (figure 5*i*). The *elf3–4* allele contains a small deletion in the coding region [50] and accumulates the mutant RNA. The mutant expression profile suggests de-repression at ZT2 (*p* = 0.06; figure 6*i*), consistent with lower expression of *LHY* and *CCA1* in *elf3* (noted above).

#### 2.5.6. *ELF4* and *LUX*

The two remaining EC components tested, *ELF4* and *LUX,* share the evening expression peak determined by *LHY/CCA1-*mediated repression, with a phase advance in *lhy;cca1* and a delay in *prr7;prr9* in LD and LL conditions (figure 5*g,j*). Strikingly, however, the phase separation among the clock genes was lost in the *lhy;cca1* double mutant under LL, such that *PRR9* and *ELF4* peaked together at 50 and 66 h (discussed below). Thus, LHY and CCA1 contribute to the 4 h separation of peak times between *PRR9* (54 h) and *ELF4* (58 h) in the Ws-2 control under LL. In DD, peak expression of *ELF4* was the most reduced of all the genes, to less than 10% of the LD peak level (*p* < 0.01 in Col and Ws; figure 5*t*), consistent with the loss of light activation [44] and/or sugar signalling. *ELF4* was also de-repressed earlier in the *toc1* mutant under DD than the other genes (ZT28–36 h; figure 5*j*), rising as early as in the *lhy;cca1* double mutant. Under LD conditions, the *toc1* mutant de-repressed *ELF4* at ZT2–6, earlier than WT. Peak expression of *LUX* did not fall significantly in DD (figure 5*q*).

*LUX* was broadly de-repressed in the *elf3* mutant, remaining at the WT peak level at ZT6–22 h (figure 6*g*), in a similar pattern to *PRR7*. This result is consistent with LUX binding to its cognate promoter [20] resulting in negative autoregulation (figure 1*a* [10]). *ELF4* expression in the *elf3* mutant, in contrast, showed a pattern more similar to *TOC1* and *PRR5* (see above)*,* with de-repression only from ZT22–ZT6 h (figure 6*j*).

### 2.6. Alternative visualization gives new insights into co-regulation of clock genes

**Figure 7.**
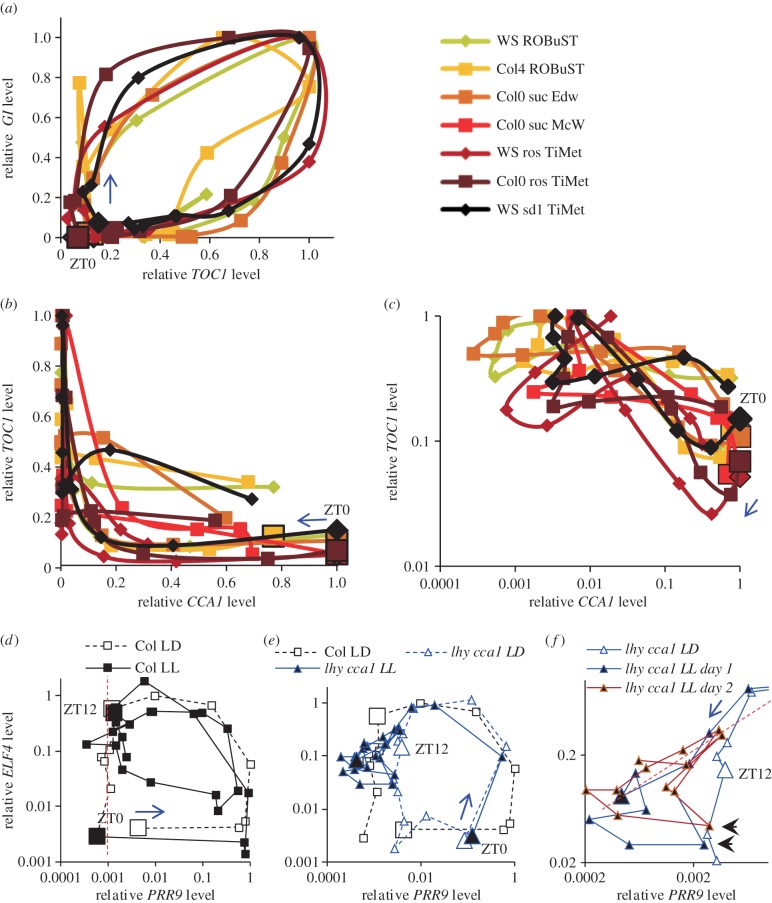
Phase plane diagrams reveal pairwise gene interactions. (*a–c*) Normalized RNA profiles of figure 3 are represented as phase plane diagrams, plotting (*a*) *GI* and *TOC1*, and *TOC1* and *CCA1* on (*b*) linear and (*c*) logarithmic scales. Larger markers indicate ZT0 datapoint, arrows indicate the direction of time. (*d–f*) RNA profiles of figure 5 are represented as phase plane diagrams on logarithmic scales, plotting data for *ELF4* and *PRR9* (*d*) in wild-type Col plants under LD and LL (0–22 h in figure 5, dashed line; 24–70 h, solid line), and (*e*) in Col plants under LD and *lhy cca1* double mutants under LD and LL (solid blue line), with (*f*) a rescaled view of a subset of the data from the *lhy cca1* double mutants. Larger markers indicate 0 (ZT0) and 12 h (ZT12) datapoints in the cycle labelled LD. These timepoints are equivalent to 24 (ZT0) and 36 h (ZT12) in the cycle labelled LL. Arrows indicate the direction of time. (*d*) Red dashed line marks falling *ELF4* levels during the night-time trough of *PRR9* in LD*.* (*f*) Red dashed line marks correlated *PRR9* and *ELF4* levels; arrowheads mark an earlier peak on each cycle in *PRR9.* Timepoints 48 (ZT24) to 70 h (ZT46) under LL are plotted in brown to emphasize the similar profiles on successive days.

Data visualization is critical in analysing the complex interactions within the clock gene circuit, in order to generate new hypotheses. Timeseries plots do not show these interactions directly. They can be revealed in phase plane diagrams that plot the levels of two components against each other (figure 7), though this format is less familiar (see electronic supplementary material). First, phase plane plots emphasize the relative timing of clock components, rather than control by the light : dark cycle. For example, *GI* rose without (before) *TOC1*, especially in Col plants of the TiMet and ROBuST datasets that were grown without exogenous sucrose. High *TOC1* levels extended later than high *GI*, particularly in Ws-2 plants of the TiMet datasets (figure 7*a*). Second, this visualization can reveal interactions among the components plotted. For example, figure 7*b* shows *TOC1* RNA levels in younger plants were maintained at 35–55% of the peak level at ZT20–22, when *CCA1* expression rose above 50% of its peak level. *TOC1* levels were lower for the same *CCA1* level in rosette plants. The logarithmic scale shows this more clearly (figure 7*c*). This suggests that CCA1 protein is not yet an effective repressor of *TOC1* at this phase, especially in younger tissues.

Finally, the phase plane diagrams can show how the interaction of two genes depends upon a third regulator. Expression peaks of *PRR9* and *ELF4* were far out of phase in the WT (figure 7*d*), for example. Data from LL (filled symbols) suggest a negative correlation in the subjective night, when *ELF4* falls as *PRR9* rises. However, the two genes peak then fall together in the *lhy cca1* double mutant under LL, at ZT26 and ZT42 (figure 7*e*; equivalent to timepoints 50 and 66 h in figure 5), creating a diagonal with a positive gradient (red dashed line, figure 7*f*). *PRR9* also had an earlier peak that was not shared by *ELF4* (ZT22 and ZT38, or 46 and 62 h in figure 5; black arrowheads in figure 7*f*). Both features were reproduced on two successive cycles, though *PRR9* expression was less than 1% of the WT peak level. Thus *LHY*, *CCA1* and the LD cycle all differentiate *PRR9* expression from *ELF4*, but in their absence, *PRR9* and *ELF4* expression profiles are similar for much of the circadian cycle (six of eight timepoints in the short, 16 h cycle of the mutant), presumably controlled by the other PRRs and/or the EC. Likewise, phase plane diagrams for the *prr7;prr9* double mutant (electronic supplementary material, figure S6) suggested that not only CCA1 and LHY, but also the PRRs repress *ELF4* in the WT. In addition to visualization, many other aspects of data management benefit significantly from online data infrastructure.

### 2.7. Online infrastructure for data sharing

**Figure 8.**
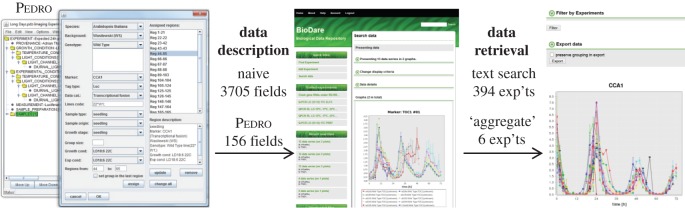
Computational infrastructure for systems chronobiology. Customized wizards in the Pedro XML editor capture detailed metadata (right panel, showing *CCA1* *:* *LUC* in sample wizard). Rather than filling 3705 metadata fields for this experiment, as a naive spreadsheet would require, Pedro captures the information with only 156 entries. After uploading the metadata and numerical data to BioDare, results can be displayed in the web browser (centre panel) with powerful secondary processing functions. The left-hand sidebar in this screen has shortcuts to common tasks and recent activity. A naive text search for ‘CCA1’ returned 394 experiments (exp'ts), whereas BioDare's ‘aggregate’ function retrieved six specific results by searching the structured metadata, with secondary filters. The search shown (right panel) aggregated qPCR assays of *CCA1* in wild-type plants (see main text) including datasets 1, 3, 4 and 6 of figure 1*c*. The export button above the graph downloads the data shown to a spreadsheet-compatible file.

Our open-source BioDare (Biological Data repository) [51] supports data from many small-scale experiments that collectively represent a significant resource (table 1). Empirical evidence indicates that these data are essential to understand complex biological regulation, and mathematical analysis shows why this is the case (see Discussion). In addition to six rhythm-analysis algorithms [52] and protocols for analysis, statistical summary and visualization [53], BioDare facilitates data sharing and public dissemination by providing a stable identifier for each experiment. Detailed metadata (experimental description) ensure that the data can be reused appropriately. Results can be compared across studies and laboratories (‘data aggregation’) by searching the metadata for genotype, marker gene and other terms (figure 8). Increased expression of *GI* in the *elf3* mutant, for example, is highlighted despite the greater technical variability of manual assay preparation in the Southern dataset compared with the later, robotized assays in the TiMet data (figure 6*h*; electronic supplementary material, figure S5*f* and Methods).

**Table 1.** Usage statistics of BioDare (Feb 2015), from originating groups and selected external users. An experiment represents a dataset similar to one of the above-described studies, which includes multiple timeseries, from samples of multiple genotypes, assays or reporters and/or environmental conditions. Totals include minor users that are not listed individually; the total number of data points is over 41 million.

research group	location	experiments	% total experiments	timeseries	% total timeseries
A. J. Millar	Edinburgh, UK	332	14	41 890	18
A. Hall	Liverpool, UK	261	11	79 228	34
D. Bell-Pedersen	Texas A&M, USA	138	6	1428	1
J. Agren	Uppsala, Sweden	18	1	9370	4
K .J. Halliday	Edinburgh, UK	230	10	5043	2
L. Larrondo	Santiago, Chile	75	3	6429	3
M. Jones	Essex, UK	89	4	3148	1
M. Hastings	MRC LMB, UK	1071	45	58 770	25
S. Harmer	UC Davis, USA	37	2	11 353	5
S. A. Kay	USC, USA	38	2	12 972	6
All BioDare		2344		232 844	

### 2.8. Optimizing clock models with public resources

One goal of such comparisons is to determine how much of the available data is matched by a particular mathematical model: the ROBuST and TiMet experiments were designed to test models of the clock gene circuit under different growth conditions. However, testing complex models against large datasets requires skills that are rare among plant molecular researchers. We therefore tested whether our comprehensive data and better computational resources could make modelling more accessible. The open-source SBSI allows non-programmers to optimize model parameters in order to match diverse data, on large, parallel computers [29]. As a test case, we addressed a recognized limitation of the original P2011 model [10], termed P2011.1.1. The model was developed to understand circadian clock function under light–dark cycles and, separately, under constant light. Following a transition from LD to LL (as in figure 5*a–j*), the first peak in expression of the combined *LHY/CCA1* component under constant light occurred at ZT28.4 h (52.4 h in figure 7*a*), about 2.5 h later than in the TiMet ros data (as noted [25,54]). The model's light–dark function was replaced with the input signal step function [55] to represent the LD–LL transition in the community model exchange format, SBML [56]. The resulting model P2011.1.2 was optimized in SBSI (see electronic supplementary material), testing model simulations with many alternative parameter sets against the TiMet ros RNA dataset, including the LD–LL transition (figure 5*a–j*), and against circadian period values for clock mutants and WT plants [29].

**Figure 9.**
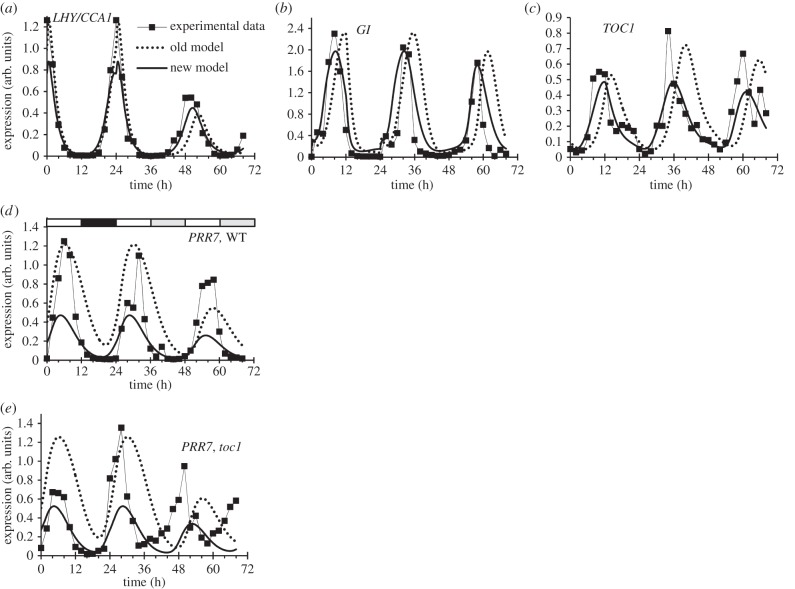
Model re-optimization. Comparison of measured transcript levels from figure 5 (experimental data, symbols), with simulation of models P2011.1.2 (old model, dotted line) and P2011.2.1 (new model, solid line), which resulted from fitting to these data using SBSI. 0–24 h, LD; 24–72 h, LL. (*a*) *LHY* and *CCA1* transcripts are combined in the model, so the average of *LHY* and *CCA1* data is plotted. The peak of *LHY/CCA1* under LL was delayed in the P2011.1.2 model (52.4 h) relative to the peak in the data (50 h), which was closely matched by the P2011.2.1 model (50.5 h). (*b*) *GI* transcript, (*c*) *TOC1* transcript and (*d*) *PRR7* transcript in Col-0 WT. (*e*) *PRR7* transcript in the *toc1* mutant shows a greater phase-advance in LL than either model. Chi-square cost value for match to TiMet ros Col-0 data in LD-LL was 20.2 for P2011.1.2, 7.6 for P2011.2.1. Chi-square cost for match to TiMet ros *toc1* data in LD-LL was 39.7 for P2011.1.2, 13.1 for P2011.2.1.

The optimized parameter set of model P2011.2.1 more closely matched the data, including an earlier peak of *LHY/CCA1* in LL at ZT26.5 h (figure 9*a*) and a closer match to *TOC1* and *GI* profiles in LD (ZT10–12 h; figure 9*b,c*), while retaining other qualitative behaviours. *LHY/CCA1* expression rises in LL after the PRR repressor proteins are degraded. Consistent with this notion, removing *TOC1*, the last gene in the *PRR* repressor wave, advanced the phase of the entire clock mechanism in LL. Results for *PRR7* are shown in figure 9*d,e*. PRR protein degradation rates were not strongly affected in P2011.2.1; rather, overall PRR levels were lower than in P2011.1.2 (not shown). In the simulated *toc1* mutant, the peak of *LHY/CCA1* was 1.4 h earlier than simulated WT in P2011.1.2, 2.5 h earlier in P2011.2.1, but 4 h earlier in the data (figure 5*a,b*). The simulations of *PRR7* show the same improved timing of the new model for the WT (figure 9*d*) and the *toc1* mutant in LD (figure 9*e*), but an earlier phase of the *toc1* mutant data under LL. Regulatory interactions among the *PRR* genes will repay further analysis [9,10] in future models (see Discussion).

The computation time required for P2011.2.1 was only approximately 30 core-hours, because the model parameters were varied within only a narrow range (two- to threefold change) from their starting values in P2011.1.2 [10]. The P2011.1.2 parameters had been manually determined to match a wide range of data and qualitative behaviours in the clock literature; many were derived from the parent model P2010 [14]. When the first model of a system is developed, in contrast, most or all parameter values may be unknown. We therefore tested our approach in such scenarios (table 2). Allowing parameter values to differ by up to 100-fold from the values in P2011.1.2 created a very large parameter space that was nonetheless centred on a known, viable region. In contrast, starting parameters from nominal values (0.1, 1, etc.) and testing each parameter over the same range (such as 0.001–10) removed that anchor. Viable parameter sets that gave cost values similar to the unmodified P2011.2.1 were identified in each test, with computation times up to four core years for P2011.6.1, using the UK national supercomputing resource HECToR. These parameter sets are not intended to replace P2011.1.2 but to demonstrate that similar results can be achieved by a more accessible approach using the TiMet data and SBSI, without new programming or laborious, manual model development. The P2011 model versions and the cognate graphical network diagram (electronic supplementary material, figure S2) are publicly accessible from the PlaSMo repository and elsewhere (see appendix A).

**Table 2.** Optimization of model parameters from loose constraints. The starting P2011.1.2 model was optimized in SBSI to fit the TiMet ros dataset and additional period constraints (see electronic supplementary material, Methods). Model, version number of the resulting model. PlaSMo ID, model identifier in the PlaSMo resource. Job, computational job code. Start, the default parameters values from P2011.1.2 or nominal values (Nom). Range, the range of parameter values that were searched, either as fold change above and below the P2011.1.2 values or as a fixed range. Set-up trials, the number of randomly chosen parameter sets tested to initialize the optimization. Cost, the best cost value (closest fit to all constraints).

model	PlaSMo ID	internal job ID	start	range	set-up trials	cost
P2011.1.2	PLM_71 ver 1	—	—	—	—	171
P2011.2.1	PLM_71 ver 2	.599	P2011.1.2	2–3×	5000	77
P2011.3.1	PLM_1041 ver 1	t30	P2011.1.2	100×	2 097 152	175
P2011.4.1	PLM_1042 ver 1	t34	Nom	0.001–10	67 108 864	270
P2011.5.1	PLM_1043 ver 1	t37	Nom	0.001–10	67 108 864	190
P2011.6.1	PLM_1044 ver 1	t40	Nom	0.0005–20	134 217 728	185

## 3. Discussion

### 3.1. Robust regulation of clock gene expression

Quantitative timeseries data are crucial to understand the dynamics of any moderately complex regulatory system. As understanding advances, more precise questions can be formulated that demand both consistent and comprehensive datasets. We provide such data for the RNA profiles of genes associated with the *Arabidopsis* circadian clock, with an online resource to facilitate comparisons within and across datasets. Our experiments were designed to test clock function under the distinct conditions required for separate studies, on light signalling (in the ROBuST project) and carbon metabolism (in the TiMet project), using different technical platforms. The results presumably include the variation previously observed among experiments designed to be replicated across laboratories [57]. We compared two *Arabidopsis* accessions. Significant differences in circadian timing have been demonstrated among *Arabidopsis* accessions, albeit using long-term, imaging assays that integrate the effects of small timing changes over many cycles [58–60]. Importantly, the rhythmic RNA profiles tested here were remarkably consistent (figure 3). Progress in understanding the clock gene network must, in part, be attributed to this robustness of circadian regulation.

Several clock genes are regulated with high daily amplitude, more than 100-fold for *LHY, CCA1, GI, ELF4* and *PRR5* under LD (figure 4; electronic supplementary material, figures S3 and S4 [21,34]), falling to low RNA copy numbers per cell. Our data necessarily reflect the mean expression across cells in the rosette, greater than 80% of which are in the leaf mesophyll [61]. Nonetheless, the absolute calibration of our RNA assays provides one approach for future estimation of the average copy number for the cognate proteins.

The most striking variations of RNA profiles among WT plants involved the acutely light-responsive genes *GI* and *PRR9*. The ROBuST dataset showed the highest levels of *GI* and strong induction of *PRR9* at ZT2 (figures 2 and 3). This is consistent with strong light induction, which might be mediated by direct photoreceptor signalling and/or by indirect sugar signalling. The absence of exogenous sucrose in the ROBuST conditions was not the sole cause, as the TiMet sd2 data used the same, sucrose-free media but did not show such strong *GI* induction (figure 3*c*). The lower growth temperature in ROBuST conditions (17°C rather than 20–22°C in other datasets) might also increase light responsiveness. Consistent with this notion, both exogenous sucrose and higher ambient temperature limit other light responses [30,31].

### 3.2. Regulation of the *PRR* repressors

RNA profiles of the *PRR* gene family varied among datasets in the WT under LD, as well as among conditions and genotypes. The variable expression of *TOC1* around ZT18 (figure 3*b*) awaits a mechanistic explanation, as do the de-repression of multiple genes in DD (for example, figure 5*n*) and of *PRR5* in the *gi* mutant (figure 5*e*; electronic supplementary material, figure S5*b*). TOC1 is thought to be an active repressor at ZT18, so variable auto-repression is possible and might also explain variation in *PRR5* expression at this phase (figure 2*g*). Alternatively, *TOC1* expression might rise during a transition between one repressor in the early night (such as the EC) and another in the late night (such as LHY and CCA1).

The tight interconnections among the clock components complicate the analysis of these data, though the resulting combination of direct and indirect effects is now interpretable. For example, removing EC regulation in the *elf3* mutant de-repressed the direct EC targets *PRR9* and *PRR7* in the early night, when the EC is active in WT plants. *PRR5* and *TOC1* were noted as potential targets based on mutant RNA profiles [10], but both genes were de-repressed around dawn in *elf3*, suggesting that an indirect mechanism owing to lower LHY and CCA1 levels is more significant than the loss of direct regulation by the EC in the mutant. *PRR9* and *PRR7* are both proposed EC targets (along with *ELF4* and *LUX*), yet *PRR9* (and *ELF4*) retains rhythmic regulation in the *elf3* mutant under LD, whereas *PRR7* (and *LUX*) is more severely affected (figure 6). To understand such differences in response, it will now be important to measure the affinity of regulators for their target genes, extending initial data [62]. Previous modelling results indicated that the different daily profiles of the *PRR* genes allow flexible responses to dawn and dusk [14], so the mechanisms that generate the *PRR* profiles will repay further analysis [10,11].

Several results suggested that regulation by the *PRR* genes is light-dependent. First, in the *prr7;prr9* double mutant, *LHY* and *CCA1* expression was de-repressed during the day but returned to match the WT profile at night in LD (figure 5). One explanation might be that PRR9 and PRR7 (directly or indirectly) antagonize the light activation of *LHY* and *CCA1* during the day in the WT [14,63], and the absence of these PRR proteins in the double mutant has little effect in darkness. Consistent with this notion, the *prr9* single mutant also showed a day-time de-repression of *CCA1* in the ROBuST dataset (electronic supplementary material, figure S5*e*), albeit less than in the double mutant. However, the *CCA1* profile in the *prr7* single mutant was unaffected in the daytime, but de-repressed 2 h earlier in the night (electronic supplementary material, figure S5*e*). Thus, inter-regulation of the early *PRR* genes is important, in addition to regulation by *TOC1* [10]. Second, in the *lhy;cca1* double mutant, *PRR* gene expression is repressed to low levels at the end of the day in LD, consistent with simultaneous, early expression of all the PRR repressors in these mutant plants. In DD, however, the falling phase of *PRR* expression is the same in WT and double mutant plants (figure 5). The higher and earlier expression of the *PRR* RNAs in the double mutant in DD does not appear to be effective in suppressing *PRR* expression. The faster degradation of the PRR proteins in darkness presumably contributes to these effects; it will be interesting to determine whether the interaction of the photoreceptor PHYB with clock proteins (including TOC1 [64]) also mediates the light sensitivity of this process.

### 3.3. Effects of exogenous sucrose

Current models of the *Arabidopsis* circadian clock are necessarily based on disparate data, much of it derived from seedlings grown on media containing high levels of sucrose. The presence or absence of exogenous sucrose under the conditions tested here affected the clock RNA profiles less, or at least no more, than other experimental parameters, despite the widespread regulation of plant genes by sucrose [41,65]. Consistent with this, effects of exogenous sucrose on clock gene expression in WT plants have previously been reported under very low light fluence rates or in the presence of photosynthetic inhibitors [40], in DD, CO_2_-free air or the starchless *pgm* mutant [39,66,67]. *PRR7* was induced in sugar-starved conditions (extended DD and at night in *pgm*) and was repressed by resupply of 3% exogenous sucrose (electronic supplementary material, figure S7*a*). Only the TiMet rosette study tested *PRR7* in DD, finding increased *PRR7* levels (figure 5*n*), especially in the trough of the profile (electronic supplementary material, figure S4*c*). Trough levels of *CCA1* and *GI* were also raised in DD in the TiMet data, and in the Edwards experiment that included 3% exogenous sucrose (electronic supplementary material, figure S3 [34]). De-repression of the trough levels in DD is neither specific to *PRR7* nor to sugar limitation. Transcript levels of the TOC1- and PRR5-degrading F-box protein ZTL, and its homologues LKP2 and FKF1, also rose slightly in sugar-starved conditions (electronic supplementary material, figure S7*b* [67]), suggesting one possible mechanism for de-repression of *PRR7* via faster degradation of PRR repressors.

### 3.4. Open resources for small-scale results

Our results will be useful to generate and test many hypotheses beyond those reported here. The potential for such future value might, in principle, justify the additional effort in curating and disseminating our data. In practice, future value motivated little data sharing, compared with present value. We therefore outline the mathematical understanding of and empirical evidence for such present value, together with practical steps that increased both present and future value relative to the effort involved in sharing data.

No suitable community repository existed for our results. One reason was the relatively large effort required to describe accurately many small data files, which deters researchers and resource developers from sharing such data [68]. The largest-scale omics and sequencing studies have different data structures, motivations, stakeholders and economics, which can facilitate data sharing [69] including exemplary resources in the circadian field [70–72]. However, mathematical analysis explains why the results of small-scale experiments are often particularly valuable in understanding biological systems. Gutenkunst *et al.* [73] showed that parameters were ‘sloppy’ in dynamic models of a range of biological regulatory systems, meaning that a wide range of parameter values could generate the simple behaviours that they tested. Rand *et al.* [74–76] tested how many parameter changes could affect the dynamic behaviour of such systems. All possible behaviours were tested and only a handful of behaviours could be readily achieved by changing parameters (these behaviours have also been termed the ‘dynatype’ of the system, by analogy to the phenotype of an organism [77]). For circadian clocks, a change in period was the most accessible behaviour: many different parameter changes altered period under constant conditions [74]. The related, empirical result is that genetic screens seeking mutants with altered circadian period have not only identified clock components, but also many genes that affect the clock less directly [78,79]. Observing a change in period gives little evidence for the role of the mutated gene in the plant and does not strongly constrain any particular parameter in the model, but rather has a small constraining effect upon a large number of parameters, in agreement with Gutenkunst *et al.* [73]. A measured period value can therefore easily be accommodated without fundamentally changing the model. In contrast, manipulating the system to test less accessible behaviours provides strong constraints, albeit potentially on fewer parameters [76]. It is much more likely that such results would not be accommodated by any reasonable parameter values, falsifying the current model and leading to new understanding during the development of a better model. Thus, the number of manipulations tested is crucial; model analysis can prioritize the most informative manipulations [80,81].

One consequence for experimental design is that the number of manipulations is more important than the number of components tested. This concept is familiar from the statistical clustering of microarray timeseries. The behaviour of a single cluster mean can adequately represent hundreds of individual transcripts, even for genes with complex light and circadian regulation [82]. The individual transcript data are more valuable in identifying coregulated, downstream genes than in understanding the clock system upstream. Thus, targeted qRT-PCR or reporter gene assays have been more widely used in understanding the clock gene circuit, although they lacked a data-sharing resource. Despite the limited justification for costly omic assays, targeted data-sharing resources [67,70] have ensured that a subset of transcriptomics data have been reused effectively in clock studies.

Empirical evidence for the value of multiple manipulations comes from 10 years of modelling the plant clock gene circuit and output pathways. Constraining the models with timeseries data from many conditions was a critical tool [83], resulting in multiple, experimentally validated predictions. Gleaning the data from electronic supplementary material or by ‘scraping’ numerical values from published charts made this possible. In practice, aggregating the numerical data has often taken a major effort, after which the data were shared on author web sites [25,84] or on BioDare [82,85,86].

BioDare [51] was developed to share timeseries data from relatively small-scale experiments conducted within individual laboratories (such as the Edwards, Southern and McWatters datasets) or in collaborative projects with few partners (such as ROBuST and TiMet). A regular user might upload an experiment with several hundred timeseries each week [87,88]. However, the user must also provide experimental metadata that are sufficiently detailed to pinpoint the most relevant experiment among hundreds to thousands of similar studies (table 1). The resource must therefore streamline the process of writing the structured metadata to minimize the weekly effort involved, and then use the metadata to provide powerful search functions, for later users to discover relevant data that were previously unknown to them. Figure 8 illustrates metadata capture using ‘wizard’ forms in BioDare, data aggregation based upon the resulting metadata, and visualization of the data from a small set of relevant experiments, whereas a naive text search returned an impractically large number of results.

The potential future value of shared data resulted in fewer than a dozen datasets being shared in the early phases of our projects. To provide immediate value from depositing data, BioDare therefore offers data processing (detrending, averaging) and visualization along with specialized circadian data analysis [52,53]. Stable identifier URLs conveniently direct collaborators to specific datasets and can be cited in publications [88,89]. The citations will be tracked by the Thomson Reuters Data Citation Index, giving a metric analogous to publication citations to recognize data-sharing contributions [90]. BioDare is available as a community resource that could be linked to organism-specific databases [91]. BioDare complements our repository of plant systems models (PlaSMo) [92].

### 3.5. From visualization to modelling

Our analysis here was model-assisted but manual, so data visualization was important. For example, phase-plane diagrams can reveal conditional pairwise interactions including subtle effects at low RNA levels, such as the correlation of *PRR9* and *ELF4* expression in the *lhy cca1* double mutant under LL (figure 6*d–f*). In contrast, *PRR9* and *ELF4* expression are uncorrelated or anticorrelated in the WT under LD. Such changes in dynamics are important in forming hypotheses during model development. Expert modelling has a subjective element. Objective machine-learning methods can also to contribute to hypothesis generation [93], though understanding such a conditionally connected network (electronic supplementary material, figure S2) is challenging by any approach [94].

Dense transcriptional regulatory interactions might be general for plant environmental response pathways [95], justifying investment in infrastructure to support their analysis. Mathematical models can powerfully express hypotheses about such circuits, so long as the starting model adequately recapitulates most data. Qualitatively, the variation among our datasets was smaller than the departure of the model simulations from the data (figures 3 and 9). The existing circadian clock models are therefore equally applicable to the several growth conditions tested, at least in leaf tissue.

The transition from LD to LL is one case where the model departed from the data, to which it had not previously been constrained (also noted in references [54,88]). The P2011.2.1 model's 2 h late phase in LL (figure 9) is caused by the slower degradation of PRR proteins in the light than in the dark [16,45]. Without a dark night to reduce PRR levels, their slow degradation delays the rise in *LHY/CCA1* on the first cycle in LL in the model. *PRR9, PRR7* and *PRR5* RNA levels are reduced in the second cycle in LL in both model and data (figures 5*c–e* and 9*e*), restoring an approximately 24 h period in subsequent cycles. It is reassuring but not surprising that re-optimization of the model could better match this behaviour, but the models' detailed behaviour is non-trivial. Reducing the levels of PRR proteins in the new parameter set advanced the phase of the first peak in LL. Simplified models that included only the PRR protein changes also reduced the effect of the PRRs on the period of the clock in constant light (data not shown), contradicting the data. The re-optimization allowed multiple parameter changes to advance the phase of the P2011.2.1 model under LL while retaining the observed effects of PRRs on clock period, such as the short period of the *toc1* mutant (figure 9*e*).

Most significantly, this result was obtained using tools designed to be accessible to biological researchers with no specialist computing or mathematical skills. Development of P2011.2.1 required no new programming, nor the hand-crafted cost functions that were used to optimize previous models [25,83–85], nor the laborious, expert parameter exploration used to construct its parent models [10,14,96]. Our intention was that the scarcity of these skills should no longer present an insuperable barrier, though of course they remain beneficial, not least to keep abreast of relevant method development [80]. To test whether this approach could assist new model development, as well as adjustment of an existing model, we repeated the parameter search within a wide range of values and/or after setting P2011.1.2 model parameters to nominal values. Greater computational power is required when there are fewer constraints on the model's parameter values; however, viable solutions were identified (table 2) and suitable computing resources are increasingly accessible [97]. The approach and infrastructure presented here allow a wider range of biologists to engage with complicated models, which will be essential tools to understand the mechanisms and physiological functions of complex biological networks.

## Supplementary Material

Supporting Information and Supplementary Figures

## Appendix A

**A.1. Experimental procedures**

Experimental methods were similar or identical to published protocols [33,88,98], as detailed in the electronic supplementary material. Statistical significance of comparisons reported in the results is from two-tailed *t*-tests compared with the cognate WT plants at the same timepoint, unless otherwise stated. Homoscedasticity is assumed, because all comparisons reported are within individual datasets for the same PCR primers. Significance is not corrected for multiple comparisons (reducing significance), nor for support from neighbouring timepoints or replication across cycles or studies (which can increase significance).

**A.2. Biodare and computational methods**

The BioDare online resource (www.biodare.ed.ac.uk) uses a desktop application to prepare metadata (describe experiments). The XML editor Pedro [99] was customized for each experimental protocol to speed up metadata entry, as each experiment can comprise several hundred samples. Numerical data are uploaded in a spreadsheet-compatible format, with the XML metadata, and stored in a relational database. Password-protected access allows controlled data sharing or public dissemination. Searching the metadata (by genotype, marker, etc.) allows aggregation of similar data from multiple sources, followed by secondary processing (detrending, normalization, averaging), visualization (figure 7*a*) and download. Rhythm analysis in BioDare was recently described [52,53]. Model optimization used SBSIvisual v. 1.4.5 [29] and SBSInumerics v. 1.2 (see electronic supplementary material, Methods). Graphical network diagrams used SBGN-ED in VANTED [38].

**A.3. Data, network diagram, model and code accessibility**

The accessibility of resources used in the publication is summarized at the University of Edinburgh's institutional repository, at http://www.research.ed.ac.uk/portal/en/datasets/data-code-and-models-for-flis-et-al-rs-open-biology-2015(fd297498-7d0d-4d57-9040-769af9c65212).html

**A.3.1. RNA expression profile data**

The RNA datasets reported here are publicly available from BioDare with the permanent data identifiers listed below, using login name ‘public’ with password ‘public’. Numbers below match figure 1.

(1) Pinas-Fernandez and K.J. Halliday (2015) ROBuST RNA timeseries data at 17°C for clock model parameterisation. BioDare accessions:

ROBuST sd for *CCA1*, BioDare accession 12820611467827, https://www.biodare.ed.ac.uk/robust/ShowExperiment.action?experimentId=12820611467827

ROBuST sd for *LHY*, BioDare accession 3492, https://www.biodare.ed.ac.uk/robust/ShowExperiment.action?experimentId=3492

ROBuST sd for *PRR9*, BioDare accession 12820610743262, https://www.biodare.ed.ac.uk/robust/ShowExperiment.action?experimentId=12820610743262

ROBuST sd for *PRR7*, BioDare accession 12820611319996, https://www.biodare.ed.ac.uk/robust/ShowExperiment.action?experimentId=12820611319996

ROBuST sd for *PRR5*, BioDare accession 12820611188065, https://www.biodare.ed.ac.uk/robust/ShowExperiment.action?experimentId=12820611188065

ROBuST sd for *TOC1*, BioDare accession 12820611587928, https://www.biodare.ed.ac.uk/robust/ShowExperiment.action?experimentId=12820611587928

ROBuST sd for *GI*, BioDare accession 12820606741450, https://www.biodare.ed.ac.uk/robust/ShowExperiment.action?experimentId=12820606741450

ROBuST sd for *LUX*, BioDare accession 12820610913763, https://www.biodare.ed.ac.uk/robust/ShowExperiment.action?experimentId=12820610913763

ROBuST sd for *CAB2*, BioDare accession 13228354371807, https://www.biodare.ed.ac.uk/robust/ShowExperiment.action?experimentId=13228354371807

ROBuST sd for *ELF4*, BioDare accession 12962296599986, https://www.biodare.ed.ac.uk/robust/ShowExperiment.action?experimentId=12962296599986

ROBuST sd for *ELF3*, BioDare accession 12962294335805, https://www.biodare.ed.ac.uk/robust/ShowExperiment.action?experimentId=12962294335805

(2) Flis, V. Mengin, R. Sulpice and M. Stitt (2015) TiMet RNA timeseries data from rosette plants for clock model parameterisation. TiMEt ros, BioDare accession 2841, https://www.biodare.ed.ac.uk/robust/ShowExperiment.action?experimentId=2841
(3) Flis, V. Mengin, R. Sulpice and M. Stitt (2015) TiMet RNA timeseries data from *elf3* mutant plants for clock model parameterisation. TiMet sd1, BioDare accession 2842, https://www.biodare.ed.ac.uk/robust/ShowExperiment.action?experimentId=2842
(4) Flis, V. Mengin, R. Sulpice and M. Stitt (2015) TiMet RNA timeseries data from seedlings for clock model parameterisation. TiMet sd2, BioDare accession 2843, https://www.biodare.ed.ac.uk/robust/ShowExperiment.action?experimentId=2843
(5) H. G. McWatters (2015) Clock RNA timeseries in light and temperature entrainment. McWatters sd, BioDare accession 3488, https://www.biodare.ed.ac.uk/robust/ShowExperiment.action?experimentId=3488
(6) K.D. Edwards and A.J. Millar (2010). Clock RNA timeseries in multiple photoperiods. Edwards sd, BioDare accession 13227622661196, https://www.biodare.ed.ac.uk/robust/ShowExperiment.action?experimentId=13227622661196
(7) M.M. Southern and A.J. Millar (2015). Clock RNA timeseries in wild-type and mutant plants under red light.

Independent biological replicates are presented for this experiment, with the same genotypes and markers tested in replicates (1 + 2) and (3 + 4):

Southern sd replicate 1, Biodare accession 13228298288040, https://www.biodare.ed.ac.uk/robust/ShowExperiment.action?experimentId=13228298288040

Southern sd replicate 2, Biodare accession 13227619871305, https://www.biodare.ed.ac.uk/robust/ShowExperiment.action?experimentId=13227619871305

Southern sd replicate 3, Biodare accession 13228357055348, https://www.biodare.ed.ac.uk/robust/ShowExperiment.action?experimentId=13228357055348

Southern sd replicate 4, Biodare accession 13228357183121, https://www.biodare.ed.ac.uk/robust/ShowExperiment.action?experimentId=13228357183121

**A.3.2. Code**

BioDare and SBSI are open-source and available from Sourceforge (www.sourceforge.net).

BioDare: Sourceforge project, http://sourceforge.net/projects/biodare/. The online resource is available at www.biodare.ed.ac.uk.

SBSI: Sourceforge project, http://sourceforge.net/projects/sbsi/. Related materials, plugins and tutorials are available at www.sbsi.ed.ac.uk.

**A.3.3. Graphical network diagram**

The diagram of the *Arabidopsis* clock model (electronic supplementary material, figure S2) is available from the PlaSMo repository (www.plasmo.ed.ac.uk), which handles a variety of XML file formats.

D. D. Seaton and A. J. Millar (2015), Graphical network diagram of the *Arabidopsis* clock model P2011.1 in SBGN PD: PlaSMo accession 1045 version 1, http://www.plasmo.ed.ac.uk/plasmo/models/model.shtml?accession=PLM_1045&version=1

**A.3.4. Models**

A. Pokhilko *et al.* (2012), *Arabidopsis* clock model P2011.1.1 (published in Molecular Systems Biology, 2012): BioModels identifier BIOMD0000000412 [100].

The *Arabidopsis* clock models below are available from the PlaSMo repository (www.plasmo.ed.ac.uk) and will also be submitted to Biomodels when the present publication has a digital identifier. The model versioning convention is described in the electronic supplementary material.

A. J. Millar and A. Hume (2015) *Arabidopsis* clock model P2011.1.2: PlaSMo accession PLM_71 version 1, http://www.plasmo.ed.ac.uk/plasmo/models/model.shtml?accession=PLM_71&version=1

A. J. Millar and A. Hume (2015) *Arabidopsis* clock model P2011.2.1: PlaSMo accession PLM_71 version 2, http://www.plasmo.ed.ac.uk/plasmo/models/model.shtml?accession=PLM_71&version=2

K. Stratford, A. Hume and A. J. Millar (2015) *Arabidopsis* clock model P2011.3.1: PlaSMo accession PLM_1041 version 1, http://www.plasmo.ed.ac.uk/plasmo/models/model.shtml?accession=PLM_1041&version=1

K. Stratford, A. Hume and A. J. Millar (2015) *Arabidopsis* clock model P2011.4.1: PlaSMo accession PLM_1042 version 1, http://www.plasmo.ed.ac.uk/plasmo/models/model.shtml?accession=PLM_1042&version=1

K. Stratford, A. Hume and A. J. Millar (2015) *Arabidopsis* clock model P2011.5.1: PlaSMo accession PLM_1043 version 1, http://www.plasmo.ed.ac.uk/plasmo/models/model.shtml?accession=PLM_1043&version=1

K. Stratford, A. Hume and A. J. Millar (2015) *Arabidopsis* clock model P2011.6.1: PlaSMo accession PLM_1044 version 1, http://www.plasmo.ed.ac.uk/plasmo/models/model.shtml?accession=PLM_1044&version=1

## References

[1] Zhang EE, Kay SA 2010 Clocks not winding down: unravelling circadian networks. Nat. Rev. Mol. Cell Biol. 11, 764–776. (doi:10.1038/nrm2995) 20966970 10.1038/nrm2995

[2] Dong G, Golden SS 2008 How a cyanobacterium tells time. Curr. Opin. Microbiol. 11, 541–546. (doi:10.1016/j.mib.2008.10.003) 18983934 10.1016/j.mib.2008.10.003 PMC2692899

[3] Dodd AN et al. 2008 Plant circadian clocks increase photosynthesis, growth, survival, and competitive advantage. Science 309, 630–633. (doi:10.1126/science.1115581) 16040710 10.1126/science.1115581

[4] Ouyang Y, Andersson CR, Kondo T, Golden SS, Johnson CH 1998 Resonating circadian clocks enhance fitness in cyanobacteria. Proc. Natl Acad. Sci. USA 95, 8660–8664. (doi:10.1073/pnas.95.15.8660) 9671734 10.1073/pnas.95.15.8660 PMC21132

[5] Kinmonth-Schultz HA, Golembeski GS, Imaizumi T 2013 Circadian clock-regulated physiological outputs: dynamic responses in nature. Semin. Cell Dev. Biol. 24, 407–413. (doi:10.1016/j.semcdb.2013.02.006) 23435352 10.1016/j.semcdb.2013.02.006 PMC3742325

[6] Young MW, Kay SA 2001 Time zones: a comparative genetics of circadian clocks. Nat. Rev. Genet. 2, 702–715. (doi:10.1038/35088576) 11533719 10.1038/35088576

[7] van Ooijen G, Millar AJ 2012 Non-transcriptional oscillators in circadian timekeeping. Trends Biochem. Sci. 37, 484–492. (doi:10.1016/j.tibs.2012.07.006) 22917814 10.1016/j.tibs.2012.07.006

[8] Le Novere N et al. 2009 The systems biology graphical notation. Nat. Biotechnol. 27, 735–741. (doi:10.1038/nbt.1558) 19668183 10.1038/nbt.1558

[9] Pokhilko A, Mas P, Millar AJ 2013 Modelling the widespread effects of TOC1 signalling on the plant circadian clock and its outputs. BMC Syst. Biol. 7, 23 (doi:10.1186/1752-0509-7-23) 23506153 10.1186/1752-0509-7-23 PMC3614443

[10] Pokhilko A, Fernandez AP, Edwards KD, Southern MM, Halliday KJ, Millar AJ 2012 The clock gene circuit in *Arabidopsis* includes a repressilator with additional feedback loops. Mol. Syst. Biol. 8, 574 (doi:10.1038/msb.2012.6) 22395476 10.1038/msb.2012.6 PMC3321525

[11] Nakamichi N 2011 Molecular mechanisms underlying the *Arabidopsis* circadian clock. Plant Cell Physiol. 52, 1709–1718. (doi:10.1093/pcp/pcr118) 21873329 10.1093/pcp/pcr118 PMC3189347

[12] Farre EM, Harmer SL, Harmon FG, Yanovsky MJ, Kay SA 2005 Overlapping and distinct roles of PRR7 and PRR9 in the *Arabidopsis* circadian clock. Curr. Biol. 15, 47–54. (doi:10.1016/j.cub.2004.12.067) 15649364 10.1016/j.cub.2004.12.067

[13] Salome PA, McClung CR 2005 PSEUDO-RESPONSE REGULATOR 7 and 9 are partially redundant genes essential for the temperature responsiveness of the *Arabidopsis* circadian clock. Plant Cell 17, 791–803. (doi:10.1105/tpc.104.029504) 15705949 10.1105/tpc.104.029504 PMC1069699

[14] Pokhilko A, Hodge SK, Stratford K, Knox K, Edwards KD, Thomson AW, Mizuno T, Millar AJ 2010 Data assimilation constrains new connections and components in a complex, eukaryotic circadian clock model. Mol. Syst. Biol. 6, 416 (doi:10.1038/msb.2010.69) 20865009 10.1038/msb.2010.69 PMC2964123

[15] Nakamichi N, Kiba T, Kamioka M, Suzuki T, Yamashino T, Higashiyama T, Sakakibara H, Mizuno T 2012 Transcriptional repressor PRR5 directly regulates clock-output pathways. Proc. Natl Acad. Sci. USA 109, 17 123–17 128. (doi:10.1073/pnas.1205156109) 10.1073/pnas.1205156109 PMC3479524 23027938

[16] Nakamichi N, Kiba T, Henriques R, Mizuno T, Chua NH, Sakakibara H 2010 PSEUDO-RESPONSE REGULATORS 9, 7, and 5 are transcriptional repressors in the *Arabidopsis* circadian clock. Plant Cell 22, 594–605. (doi:10.1105/tpc.109.072892) 20233950 10.1105/tpc.109.072892 PMC2861452

[17] Huang W, Perez-Garcia P, Pokhilko A, Millar AJ, Antoshechkin I, Riechmann JL, Mas P 2012 Mapping the core of the *Arabidopsis* circadian clock defines the network structure of the oscillator. Science 336, 75–79. (doi:10.1126/science.1219075) 22403178 10.1126/science.1219075

[18] Gendron JM, Pruneda-Paz JL, Doherty CJ, Gross AM, Kang SE, Kay SA 2012 *Arabidopsis* circadian clock protein, TOC1, is a DNA-binding transcription factor. Proc. Natl Acad. Sci. USA 109, 3167–3172. (doi:10.1073/pnas.1200355109) 22315425 10.1073/pnas.1200355109 PMC3286946

[19] Nusinow DA, Helfer A, Hamilton EE, King JJ, Imaizumi T, Schultz TF, Farré EM, Kay SA 2011 The ELF4-ELF3-LUX complex links the circadian clock to diurnal control of hypocotyl growth. Nature 475, 398–402. (doi:10.1038/nature10182) 21753751 10.1038/nature10182 PMC3155984

[20] Helfer A, Nusinow DA, Chow BY, Gehrke AR, Bulyk ML, Kay SA 2011 LUX ARRHYTHMO encodes a nighttime repressor of circadian gene expression in the *Arabidopsis* core clock. Curr. Biol. 21, 126–133. (doi:10.1016/j.cub.2010.12.021) 21236673 10.1016/j.cub.2010.12.021 PMC3057456

[21] Dixon LE, Knox K, Kozma-Bognar L, Southern MM, Pokhilko A, Millar AJ 2011 Temporal repression of core circadian genes is mediated through EARLY FLOWERING 3 in *Arabidopsis*. Curr. Biol. 21, 120–125. (doi:10.1016/j.cub.2010.12.013) 21236675 10.1016/j.cub.2010.12.013 PMC3028277

[22] Herrero E et al. 2012 EARLY FLOWERING4 recruitment of EARLY FLOWERING3 in the nucleus sustains the *Arabidopsis* circadian clock. Plant Cell 24, 428–443. (doi:10.1105/tpc.111.093807) 22327739 10.1105/tpc.111.093807 PMC3315225

[23] Kim WY et al. 2007 ZEITLUPE is a circadian photoreceptor stabilized by GIGANTEA in blue light. Nature 449, 356–360. (doi:10.1038/nature06132) 17704763 10.1038/nature06132

[24] Bujdoso N, Davis SJ 2013 Mathematical modeling of an oscillating gene circuit to unravel the circadian clock network of *Arabidopsis thaliana*. Front. Plant Sci. 4, 3 (doi:10.3389/fpls.2013.00003) 23355842 10.3389/fpls.2013.00003 PMC3555133

[25] Fogelmark K, Troein C 2014 Rethinking transcriptional activation in the *Arabidopsis* circadian clock. PLoS Comput. Biol. 10, e1003705 (doi:10.1371/journal.pcbi.1003705) 25033214 10.1371/journal.pcbi.1003705 PMC4102396

[26] The Royal Society. 2012 Science as an open enterprise 2012. 29 June 2012. See https://royalsociety.org/~/media/policy/projects/sape/2012-06-20-saoe.pdf.

[27] Hey T, Tansley S, Tolle K (eds). 2009 *The fourth paradigm: data-intensive scientific discovery*. Redmond, WA: Microsoft Research.

[28] Bastow R, Leonelli S 2010 Sustainable digital infrastructure. Although databases and other online resources have become a central tool for biological research, their long-term support and maintenance is far from secure. EMBO Rep. 11, 730–734. (doi:10.1038/embor.2010.145) 20847740 10.1038/embor.2010.145 PMC2948195

[29] Adams R et al. 2013 SBSI: an extensible distributed software infrastructure for parameter estimation in systems biology. Bioinformatics 29, 664–665. (doi:10.1093/bioinformatics/btt023) 23329415 10.1093/bioinformatics/btt023 PMC3582266

[30] Stewart JL, Maloof JN, Nemhauser JL 2011 PIF genes mediate the effect of sucrose on seedling growth dynamics. PLoS ONE 6, e19894 (doi:10.1371/journal.pone.0019894) 21625438 10.1371/journal.pone.0019894 PMC3100310

[31] Franklin KA, Toledo-Ortiz G, Pyott DE, Halliday KJ 2014 Interaction of light and temperature signalling. J. Exp. Bot. 65, 2859–2871. (doi:10.1093/jxb/eru059) 24569036 10.1093/jxb/eru059

[32] Salvo-Chirnside E, Kane S, Kerr LE 2011 Protocol: high throughput silica-based purification of RNA from *Arabidopsis* seedlings in a 96-well format. Plant Methods 7, 40 (doi:10.1186/1746-4811-7-40) 22136293 10.1186/1746-4811-7-40 PMC3305896

[33] Piques M, Schulze WX, Hohne M, Usadel B, Gibon Y, Rohwer J, Stitt M 2009 Ribosome and transcript copy numbers, polysome occupancy and enzyme dynamics in *Arabidopsis*. Mol. Syst. Biol. 5, 314 (doi:10.1038/msb.2009.68) 19888209 10.1038/msb.2009.68 PMC2779082

[34] Edwards KD et al. 2010 Quantitative analysis of regulatory flexibility under changing environmental conditions. Mol. Syst. Biol. 6, 424 (doi:10.1038/msb.2010.81) 21045818 10.1038/msb.2010.81 PMC3010117

[35] Locke JC, Southern MM, Kozma-Bognar L, Hibberd V, Brown PE, Turner MS, Millar AJ 2005 Extension of a genetic network model by iterative experimentation and mathematical analysis. Mol. Syst. Biol. 1, 20050013 (doi:10.1038/msb4100018) 10.1038/msb4100018 PMC1681447 16729048

[36] Strayer C et al. 2000 Cloning of the *Arabidopsis* clock gene *TOC1*, an autoregulatory response regulator homolog. Science 289, 768–771. (doi:10.1126/science.289.5480.768) 10926537 10.1126/science.289.5480.768

[37] Fowler S et al. 1999 GIGANTEA: a circadian clock-controlled gene that regulates photoperiodic flowering in *Arabidopsis* and encodes a protein with several possible membrane-spanning domains. EMBO J. 18, 4679–4688. (doi:10.1093/emboj/18.17.4679) 10469647 10.1093/emboj/18.17.4679 PMC1171541

[38] Czauderna T, Klukas C, Schreiber F 2010 Editing, validating and translating of SBGN maps. Bioinformatics 26, 2340–2341. (doi:10.1093/bioinformatics/btq407) 20628075 10.1093/bioinformatics/btq407 PMC2935428

[39] Dalchau N et al. 2011 The circadian oscillator gene GIGANTEA mediates a long-term response of the *Arabidopsis* thaliana circadian clock to sucrose. Proc. Natl Acad. Sci. USA 108, 5104–5109. (doi:10.1073/pnas.1015452108) 21383174 10.1073/pnas.1015452108 PMC3064355

[40] Haydon MJ, Mielczarek O, Robertson FC, Hubbard KE, Webb AA 2013 Photosynthetic entrainment of the *Arabidopsis thaliana* circadian clock. Nature 502, 689–692. (doi:10.1038/nature12603) 24153186 10.1038/nature12603 PMC3827739

[41] Usadel B et al. 2005 Extension of the visualization tool MapMan to allow statistical analysis of arrays, display of corresponding genes, and comparison with known responses. Plant Physiol. 138, 1195–1204. (doi:10.1104/pp.105.060459) 16009995 10.1104/pp.105.060459 PMC1176394

[42] Osuna D et al. 2007 Temporal responses of transcripts, enzyme activities and metabolites after adding sucrose to carbon-deprived *Arabidopsis* seedlings. Plant J. 49, 463–491. (doi:10.1111/j.1365-313X.2006.02979.x) 17217462 10.1111/j.1365-313X.2006.02979.x

[43] Doyle MR, Davis SJ, Bastow RM, McWatters HG, Kozma-Bognar L, Nagy F, Millar AJ, Amasino RM 2002 The ELF4 gene controls circadian rhythms and flowering time in *Arabidopsis thaliana*. Nature 419, 74–77. (doi:10.1038/nature00954) 12214234 10.1038/nature00954

[44] Li G et al. 2011 Coordinated transcriptional regulation underlying the circadian clock in *Arabidopsis*. Nat. Cell Biol. 13, 616–622. (doi:10.1038/ncb2219) 21499259 10.1038/ncb2219

[45] Kiba T, Henriques R, Sakakibara H, Chua NH 2007 Targeted degradation of PSEUDO-RESPONSE REGULATOR5 by an SCFZTL complex regulates clock function and photomorphogenesis in *Arabidopsis thaliana*. Plant Cell 19, 2516–2530. (doi:10.1105/tpc.107.053033) 17693530 10.1105/tpc.107.053033 PMC2002626

[46] Yakir E, Hilman D, Hassidim M, Green RM 2007 CIRCADIAN CLOCK ASSOCIATED1 transcript stability and the entrainment of the circadian clock in *Arabidopsis*. Plant Physiol. 145, 925–932. (doi:10.1104/pp.107.103812) 17873091 10.1104/pp.107.103812 PMC2048808

[47] Alabadi D, Oyama T, Yanovsky MJ, Harmon FG, Mas P, Kay SA 2001 Reciprocal regulation between TOC1 and LHY/CCA1 within the *Arabidopsis* circadian clock. Science 293, 880–883. (doi:10.1126/science.1061320) 11486091 10.1126/science.1061320

[48] Millar AJ, Carré IA, Strayer CA, Chua NH, Kay SA 1995 Circadian clock mutants in *Arabidopsis* identified by luciferase imaging. Science 267, 1161–1163. (doi:10.1126/science.7855595) 7855595 10.1126/science.7855595

[49] Carre I, Veflingstad SR 2013 Emerging design principles in the *Arabidopsis* circadian clock. Semin. Cell Dev. Biol. 24, 393–398. (doi:10.1016/j.semcdb.2013.03.011) 23597453 10.1016/j.semcdb.2013.03.011

[50] Hicks KA, Albertson TM, Wagner DR 2001 EARLY FLOWERING3 encodes a novel protein that regulates circadian clock function and flowering in *Arabidopsis*. Plant Cell 13, 1281–1292. (doi:10.1105/tpc.13.6.1281) 11402160 10.1105/tpc.13.6.1281 PMC135582

[51] BioDare 2011 The Biological Data Repository. See www.biodare.ed.ac.uk.

[52] Zielinski T, Moore AM, Troup E, Halliday KJ, Millar AJ 2014 Strengths and limitations of period estimation methods for circadian data. PLoS ONE 9, e96462 (doi:10.1371/journal.pone.0096462) 24809473 10.1371/journal.pone.0096462 PMC4014635

[53] Moore A, Zielinski T, Millar AJ 2014 Online period estimation and determination of rhythmicity in circadian data, using the BioDare data infrastructure. In Plant circadian networks (ed. Staiger D), pp. 13–44. Clifton, NJ: Humana Press. 10.1007/978-1-4939-0700-7_2 24792042

[54] Dodd AN, Dalchau N, Gardner MJ, Baek SJ, Webb AA 2014 The circadian clock has transient plasticity of period and is required for timing of nocturnal processes in *Arabidopsis*. New Phytol. 201, 168–179. (doi:10.1111/nph.12489) 24102325 10.1111/nph.12489

[55] Adams RR, Tsorman N, Stratford K, Akman OE, Gilmore S, Juty N, Le Novere N, Millar AJ, Millar AJ 2012 The Input Signal Step Function (ISSF), a standard method to encode input signals in SBML models with software support, applied to circadian clock models. J. Biol. Rhythms 27, 328–332. (doi:10.1177/0748730412451077) 22855577 10.1177/0748730412451077 PMC3423168

[56] Hucka M et al. 2003 The systems biology markup language (SBML): a medium for representation and exchange of biochemical network models. Bioinformatics 19, 524–531. (doi:10.1093/bioinformatics/btg015) 12611808 10.1093/bioinformatics/btg015

[57] Massonnet C et al. 2010 Probing the reproducibility of leaf growth and molecular phenotypes: a comparison of three *Arabidopsis* accessions cultivated in ten laboratories. Plant Physiol. 152, 2142–2157. (doi:10.1104/pp.109.148338) 20200072 10.1104/pp.109.148338 PMC2850010

[58] Swarup K, Alonso-Blanco C, Lynn JR, Michaels SD, Amasino RM, Koornneef M, Millar AJ 1999 Natural allelic variation identifies new genes in the *Arabidopsis* circadian system. Plant J. 20, 67–77. (doi:10.1046/j.1365-313X.1999.00577.x) 10571866 10.1046/j.1365-313x.1999.00577.x

[59] Michael TP et al. 2003 Enhanced fitness conferred by naturally occurring variation in the circadian clock. Science 302, 1049–1053. (doi:10.1126/science.1082971) 14605371 10.1126/science.1082971

[60] Darrah C, Taylor BL, Edwards KD, Brown PE, Hall A, McWatters HG 2006 Analysis of phase of LUCIFERASE expression reveals novel circadian quantitative trait loci in *Arabidopsis*. Plant Physiol. 140, 1464–1474. (doi:10.1104/pp.105.074518) 16461388 10.1104/pp.105.074518 PMC1435814

[61] Wuyts N, Palauqui JC, Conejero G, Verdeil JL, Granier C, Massonnet C 2010 High-contrast three-dimensional imaging of the *Arabidopsis* leaf enables the analysis of cell dimensions in the epidermis and mesophyll. Plant Methods 6, 17 (doi:10.1186/1746-4811-6-17) 20598116 10.1186/1746-4811-6-17 PMC2909956

[62] O'Neill JS, van Ooijen G, Le Bihan T, Millar AJ 2011 Circadian clock parameter measurement: characterization of clock transcription factors using surface plasmon resonance. J. Biol. Rhythms 26, 91–98. (doi:10.1177/0748730410397465) 21454289 10.1177/0748730410397465

[63] Locke JC, Kozma-Bognar L, Gould PD, Feher B, Kevei E, Nagy F, Turner MS, Hall A, Millar AJ 2006 Experimental validation of a predicted feedback loop in the multi-oscillator clock of *Arabidopsis thaliana*. Mol. Syst. Biol. 2, 59 (doi:10.1038/msb4100102) 17102804 10.1038/msb4100102 PMC1682024

[64] Yeom M, Kim H, Lim J, Shin AY, Hong S, Kim JI, Nam HG 2014 How do phytochromes transmit the light quality information to the circadian clock in *Arabidopsis*? Mol. Plant. 7, 1701–1704. (doi:10.1093/mp/ssu086) 25095795 10.1093/mp/ssu086

[65] Price J, Laxmi A, St Martin SK, Jang JC 2004 Global transcription profiling reveals multiple sugar signal transduction mechanisms in *Arabidopsis*. Plant Cell 16, 2128–2150. (doi:10.1105/tpc.104.022616) 15273295 10.1105/tpc.104.022616 PMC519203

[66] Blasing OE et al. 2005 Sugars and circadian regulation make major contributions to the global regulation of diurnal gene expression in *Arabidopsis*. Plant Cell 17, 3257–3281. (doi:10.1105/tpc.105.035261) 16299223 10.1105/tpc.105.035261 PMC1315368

[67] Usadel B, Blasing OE, Gibon Y, Retzlaff K, Hohne M, Gunther M, Stitt M 2008 Global transcript levels respond to small changes of the carbon status during progressive exhaustion of carbohydrates in *Arabidopsis* rosettes. Plant Physiol. 146, 1834–1861. (doi:10.1104/pp.107.115592) 18305208 10.1104/pp.107.115592 PMC2287354

[68] Leonelli S, Smirnoff N, Moore J, Cook C, Bastow R 2013 Making open data work for plant scientists. J. Exp. Bot. 64, 4109–4117. (doi:10.1093/jxb/ert273) 24043847 10.1093/jxb/ert273 PMC3808334

[69] Toronto International Data Release Workshop A et al. 2009 Prepublication data sharing. Nature 461, 168–170. (doi:10.1038/461168a) 19741685 10.1038/461168a PMC3073843

[70] Mockler TC, Michael TP, Priest HD, Shen R, Sullivan CM, Givan SA, McEntee C, Kay SA, Chory J 2007 The DIURNAL project: diurnal and circadian expression profiling, model-based pattern matching, and promoter analysis. Cold Spring Harb. Symp. Quant. Biol. 72, 353–363. (doi:10.1101/sqb.2007.72.006) 18419293 10.1101/sqb.2007.72.006

[71] Zhang EE et al. 2009 A genome-wide RNAi screen for modifiers of the circadian clock in human cells. Cell 139, 199–210. (doi:10.1016/j.cell.2009.08.031) 19765810 10.1016/j.cell.2009.08.031 PMC2777987

[72] Patel VR, Eckel-Mahan K, Sassone-Corsi P, Baldi P 2012 CircadiOmics: integrating circadian genomics, transcriptomics, proteomics and metabolomics. Nat. Methods 9, 772–773. (doi:10.1038/nmeth.2111) 22847108 10.1038/nmeth.2111

[73] Gutenkunst RN, Waterfall JJ, Casey FP, Brown KS, Myers CR, Sethna JP 2007 Universally sloppy parameter sensitivities in systems biology models. PLoS Comput. Biol. 3, 1871–1878. (doi:10.1371/journal.pcbi.0030189) 17922568 10.1371/journal.pcbi.0030189 PMC2000971

[74] Rand DA, Shulgin BV, Salazar D, Millar AJ 2004 Design principles underlying circadian clocks. Interface 1, 119–130. 16849158 10.1098/rsif.2004.0014 PMC1618932

[75] Rand DA, Shulgin BV, Salazar JD, Millar AJ 2006 Uncovering the design principles of circadian clocks: mathematical analysis of flexibility and evolutionary goals. J. Theor. Biol. 238, 616–635. (doi:10.1016/j.jtbi.2005.06.026) 16111710 10.1016/j.jtbi.2005.06.026

[76] Rand DA 2008 Mapping global sensitivity of cellular network dynamics: sensitivity heat maps and a global summation law. J. R. Soc. Interface 5(Suppl 1), S59–S69. (doi:10.1098/rsif.2008.0084.focus) 18482906 10.1098/rsif.2008.0084.focus PMC2706458

[77] Daniels BC, Chen YJ, Sethna JP, Gutenkunst RN, Myers CR 2008 Sloppiness, robustness, and evolvability in systems biology. Curr. Opin. Biotechnol. 19, 389–395. (doi:10.1016/j.copbio.2008.06.008) 18620054 10.1016/j.copbio.2008.06.008

[78] Dunlap JC 1993 Genetic analysis of circadian clocks. Annu. Rev. Physiol. 55, 683–728. 8466189 10.1146/annurev.ph.55.030193.003343

[79] McWatters HG, Devlin PF 2011 Timing in plants: a rhythmic arrangement. FEBS Lett. 585, 1474–1484. (doi:10.1016/j.febslet.2011.03.051) 21453701 10.1016/j.febslet.2011.03.051

[80] Transtrum MK, Machta BB, Sethna JP 2010 Why are nonlinear fits to data so challenging? Phys. Rev. Lett. 104, 060201 (doi:10.1103/PhysRevLett.104.060201) 20366807 10.1103/PhysRevLett.104.060201

[81] Domijan M, Rand DA 2015 Using constraints and their value for optimization of large ODE systems. J. R. Soc. Interface 12, 20141303 (doi:10.1098/rsif.2014.1303) 25673300 10.1098/rsif.2014.1303 PMC4345496

[82] Seaton DD et al. 2015 Linked circadian outputs control elongation growth and flowering in response to photoperiod and temperature. Mol. Syst. Biol. 11, 776 (doi:10.15252/msb.20145766) 25600997 10.15252/msb.20145766 PMC4332151

[83] Locke JC, Millar AJ, Turner MS 2005 Modelling genetic networks with noisy and varied experimental data: the circadian clock in *Arabidopsis thaliana*. J. Theor. Biol. 234, 383–393. (doi:10.1016/j.jtbi.2004.11.038) 15784272 10.1016/j.jtbi.2004.11.038

[84] Salazar JD, Saithong T, Brown PE, Foreman J, Locke JC, Halliday KJ, Carré IA, Rand DA, Millar AJ 2009 Prediction of photoperiodic regulators from quantitative gene circuit models. Cell 139, 1170–1179. (doi:10.1016/j.cell.2009.11.029) 20005809 10.1016/j.cell.2009.11.029

[85] Troein C, Corellou F, Dixon LE, van Ooijen G, O'Neill JS, Bouget FY, Millar AJ 2011 Multiple light inputs to a simple clock circuit allow complex biological rhythms. Plant J. 66, 375–385. (doi:10.1111/j.1365-313X.2011.04489.x) 21219507 10.1111/j.1365-313X.2011.04489.x PMC3130137

[86] Song YH, Smith RW, To BJ, Millar AJ, Imaizumi T 2012 FKF1 conveys timing information for CONSTANS stabilization in photoperiodic flowering. Science 336, 1045–1049. (doi:10.1126/science.1219644) 22628657 10.1126/science.1219644 PMC3737243

[87] Brancaccio M, Maywood ES, Chesham JE, Loudon AS, Hastings MH 2013 A Gq-Ca^2+^ axis controls circuit-level encoding of circadian time in the suprachiasmatic nucleus. Neuron 78, 714–728. (doi:10.1016/j.neuron.2013.03.011) 23623697 10.1016/j.neuron.2013.03.011 PMC3666084

[88] Gould PD et al. 2013 Network balance via CRY signalling controls the *Arabidopsis* circadian clock over ambient temperatures. Mol. Syst. Biol. 9, 650 (doi:10.1038/msb.2013.7) 23511208 10.1038/msb.2013.7 PMC3619941

[89] Dixon LE, Hodge SK, van Ooijen G, Troein C, Akman OE, Millar AJ 2014 Light and circadian regulation of clock components aids flexible responses to environmental signals. New Phytol. 203, 568–577. (doi:10.1111/nph.12853) 24842166 10.1111/nph.12853 PMC4286021

[90] Force MM, Robinson NJ 2014 Encouraging data citation and discovery with the data citation index. J. Compt-aided Mol. Des. 28, 1043–1048. (doi:10.1007/s10822-014-9768-5) 10.1007/s10822-014-9768-5 24980647

[91] International Arabidopsis Informatics Consortium. 2012 Taking the next step: building an *Arabidopsis* information portal. The Plant Cell 24, 2248–2256. (doi:10.1105/tpc.112.100669) 22751211 10.1105/tpc.112.100669 PMC3406920

[92] PlaSMo 2010 The Plant Systems Modelling portal See www.plasmo.ed.ac.uk.

[93] Akman OE, Watterson S, Parton A, Binns N, Millar AJ, Ghazal P 2012 Digital clocks: simple Boolean models can quantitatively describe circadian systems. J. R. Soc. Interface 9, 2365–2382. (doi:10.1098/rsif.2012.0080) 22499125 10.1098/rsif.2012.0080 PMC3405750

[94] Aderhold A, Husmeier D, Grzegorczyk M 2014 Statistical inference of regulatory networks for circadian regulation. Stat. Appl. Genet. Mol. Biol. 13, 227–273. (doi:10.1515/sagmb-2013-0051) 24864301 10.1515/sagmb-2013-0051

[95] Carrera J, Rodrigo G, Jaramillo A, Elena SF 2009 Reverse-engineering the *Arabidopsis thaliana* transcriptional network under changing environmental conditions. Genome Biol. 10, R96 (doi:10.1186/gb-2009-10-9-r96) 19754933 10.1186/gb-2009-10-9-r96 PMC2768985

[96] Pokhilko A, Ramos JA, Holtan H, Maszle DR, Khanna R, Millar AJ 2011 Ubiquitin ligase switch in plant photomorphogenesis: a hypothesis. J. Theor. Biol. 270, 31–41. (doi:10.1016/j.jtbi.2010.11.021) 21093457 10.1016/j.jtbi.2010.11.021 PMC3021735

[97] Goff SA et al. 2011 The iPlant collaborative: cyberinfrastructure for plant biology. Front. Plant. Sci. 2, 34. 22645531 10.3389/fpls.2011.00034 PMC3355756

[98] Knight H, Thomson AJ, McWatters HG 2008 Sensitive to freezing integrates cellular and environmental inputs to the plant circadian clock. Plant Physiol. 148, 293–303. (doi:10.1104/pp.108.123901) 18614706 10.1104/pp.108.123901 PMC2528108

[99] Garwood KL, Taylor CF, Runte KJ, Brass A, Oliver SG, Paton NW 2004 Pedro: a configurable data entry tool for XML. Bioinformatics 20, 2463–2465. (doi:10.1093/bioinformatics/bth251) 15073025 10.1093/bioinformatics/bth251

[100] Le Novere N et al. 2006 BioModels Database: a free, centralized database of curated, published, quantitative kinetic models of biochemical and cellular systems. Nucleic Acids Res. 34, D689–D691. (doi:10.1093/nar/gkj092) 16381960 10.1093/nar/gkj092 PMC1347454

