# Supplementary material for: Defining the robust behaviour of the plant clock gene circuit with absolute RNA timeseries and open infrastructure
Source: Open Biol. 2015 Oct 14;5(10):150042. doi: 10.1098/rsob.150042 (PMC4632509; doi:10.1098/rsob.150042)
Supplement: Supporting Information and Supplementary Figures [file rsob150042supp1.pdf]

Supporting Information

Contents

Supporting Information 1

Supplementary Methods 2

Plant materials 2

Growth, harvesting, RNA extraction and analysis 2

Evaluation of datasets 5

Supplementary Analysis 6

Interpretation of Phase-Plane Diagrams 6

Model versioning and optimisation 7

Supplementary Figure Legends 8

Supplementary Figure 1. Overview of the clock gene circuit 8

Supplementary Figure 2. Detailed schema of the P2011 model 9

Supplementary Figure 3. Logarithmic plots of clock gene expression in wild-type plants under LD cycles 9

Supplementary Figure 4. RNA profiles under DD 9

Supplementary Figure 5. Mutant effects are consistent among data sets 9

Supplementary Figure 6. Phase plane diagrams reveal altered regulation in *prr7 prr9* mutants. 10

Supplementary Figure 7. Regulation of clock-related genes in low-sugar conditions 10

Supplementary Table Legends 10

Supplementary Table 1. mRNA calibration standards 10

Supplementary Table 2. qRT-PCR primer sequences 11

Supplementary References 12

Supplementary Tables 13

Supplementary Table 1 13

Supplementary Table 2. qRT-PCR primer sequences 14

Supplementary Figures 17

Supplementary Methods

Plant materials

The biological materials and allele numbers used in each experiment are shown in Figure 1C.

Growth, harvesting, RNA extraction and analysis

ROBuST dataset (Experiment 1 of Figure 1C)

Seedlings were grown on half-strength Murashige & Skoog media (MS) with 1.2 % agar, without added sucrose. Following 7 days of entrainment under LD cycles of 12h L:12h D at 22°C, seedlings were transferred to 17°C on the same light regime, and on the fifth day at 17°C seedlings were harvested every two hours into 1 ml of RNAlater (Ambion; Austin, Texas, USA). Lighting was 60 μmol/m^2/s white fluorescent light provided in a Binder (Tuttlingen, Germany) temperature-controlled incubator. Biological triplicates were independently sampled. Samples at ZT0 were taken just before lights-on, and samples at ZT12 just after lights-off. All sampling during lights-off was conduction in a temperature-controlled dark room. Total RNA was extracted (GE Healthcare Illustra RNAspin 96 RNA Isolation Kit, Buckinghamshire, UK) according to the manufacturer's instructions. cDNA was synthesized from 1 μg of total RNA (Invitrogen SuperScript VILO cDNA Synthesis kit, Paisley, Scotland) and diluted 1:10 in H_2O. qRT-PCR reactions were carried out and analysed as previously described (1) in technical triplicate. Expression values were normalized to the control transcript ACTIN 7 (ACT7), amplified with primers 5' CAGTGTCTGGATCGGAGGAT 3' and 5' TGAACAATCGATGGACCTGA 3'.

TiMet datasets

a. Plants grown on soil: TiMet rosette and seedling 1 datasets (Experiments 2 and 3 of Figure1C)

Arabidopsis thaliana seeds were sown on wet soil, covered with transparent lids and transferred to growth chambers. Plants were grown in 12 h light / 12 h dark cycles with a light intensity adjusted to 160 μmol $m^{-2}s^{-1}$. Temperature was 20℃ during the light phase and 18℃ during the dark phase. Pots were randomized to decrease positional effect. After a week lids were removed and the excess plants were thinned. After another seven days plants were treated with Nematodes as a biological pest control.

On the 21st day (rosette plants) or 13th day (seedlings) after sowing two biological replicates per time point were harvested and immediately frozen in liquid nitrogen. Each replicate consisted of a pool of 5-7 plants. Sampling was performed with 2h intervals, starting at ZT0, within 5-10 minutes before the given time point. Plant material was ground using a Ball-Mill (Retch, Germany). Around 50 mg of

material from each sample was aliquoted into 2ml microcentrifuge tubes (Eppendorf, Germany). RNA was extracted using the RNeasy Plant Mini Kit (QIAGEN) by following the manufacturer's instructions. Briefly, the RLC buffer (500µL) was added to the frozen plant powder and the mixture was homogenised. RNA standards were added (see below). RNA was eluted from the RNeasy spin column twice, first with 50 µL and then with 30 µL of RNase-free water. The concentration of RNA was determined using the Nano-Drop ND-1000 UV-Vis spectrophotometer (Nano-Drop Technologies).

b. Plants grown in sterile media: TiMet seedling 2 dataset (Experiment 4 of Figure1C)

Surface-sterilized seeds were sown on half-strength Murashige-Skoog media (MS) with 1.2 % agar without addition of sucrose. After 4 days of stratification in the dark at 4℃ plates were transferred to growth chambers and grown for 10 days in 12 light / 12 dark cycles, with light intensity of approx. 100 µmol $m^{-2}s^{-1}$ and temperature of 22℃ during the day and 18℃ during the night. Seedlings were harvested on the 10th day into 2ml Eppendorf tubes and immediately frozen in liquid nitrogen. For each time point, three independent biological replicates were collected; each included 10-20 seedlings. Samples were taken within 10 – 15 min before the given time point. Frozen seedlings were ground using a Ball-Mill (Retch, Germany). RNA was extracted using the RNeasy Plant Mini Kit (QIAGEN) but using half the volume of all buffers. RNA standards were added (see below). In the last step RNA was eluted from the RNeasy spin column once with 20 µL of RNase-free water. The concentration of RNA was determined using the Nano-Drop ND-1000 UV-Vis spectrophotometer (Nano-Drop Technologies).

c. cDNA preparation and analysis

Contaminating DNA was removed from samples using TURBO DNA-free™ kit (Applied Biosystems) by following the manufacturer's instructions. The concentration of RNA was determined using Nano-Drop spectrophotometer. 1µg of DNase treated RNA was used for cDNA synthesis. Reverse transcription used the SuperScript III First-Standard Synthesis System Kit (Invitrogen). Briefly, 1µg of DNase treated RNA was mixed with 2µL of oligo(dT) primers (50µM), 2µL of random hexamers (50ng/µL) and 1µL of dNTP mix (10mM). Mixture of a final volume of 11µL was incubated for 5 min at 65˚C in a Verti 96 Well Thermal Cycler (Applied Biosystems, Deutschland). After cooling down the mixture for 5 min on ice the extension reaction was performed by adding 0.5µL of reverse transcriptase (SuperScript III RT 200 U/µl), 2µL of 10X First strand synthesis buffer, 4µL of $MgCl_2$ (25mM), 2µL of DTT (0.1M) and 0.5µL of RNase OUT (40 U/µL). Incubation for 10 min at 25˚C was followed by 50 min incubation at 50˚C. Next, enzyme was inactivated by heating for 5 min at 85˚C. To digest all RNA that could potentially be left in samples 1 µL RNase H (2 U/µL) was added to the mixture and incubated at 37˚C for 20 min and then for 5 min at 85˚C. Samples were diluted in RNase-free water (dilution 1/5).

The cDNA was used for qRT-PCR reactions to monitor the expression level of 10 core clock genes (*LHY*, *CCA1*, *TOC1*, *PRR9*, *PRR7*, *PRR5*, *GI, ELF3, ELF4* and *LUX*). To control the quality of performed reactions, four pairs of primers encoding housekeeping genes (*ACT2 155*, *ACT2 633, GAPDH 3', GAPDH 5'*) were included to the measurements. Primer sequences are listed in Supplementary Table 2. To allow absolute quantification, 8 pairs of primers from the ArrayControl *RNA Spikes* (Applied Biosystems) were included (see below). The 22 pairs of primers were dispensed (200nM of each primer) into 384-well plates for using PerkinElmer Evolution P3 Precision Pipetting Platform (PerkinElmer Life Science, Rodgau-Jügesheim, Germany). PCR mix was prepared by adding 0.5µL of cDNA and 2.5µL of Power SYBR Green PCR Master Mix (Applied Biosystems, Deutschland) to already prepared primers. The qRT-PCR reactions were carried out following the recommended thermal profile: 2 min at 50˚C, 10 min at 95˚C, followed by 40 cycles of 95˚C for 15 s and 60 ˚C for 1 min, in an ABI PRISM 7900 HT sequence detection system (Applied Biosystems Deutschland, Darmstadt, Germany). The specificity of the amplifications was tested by heating from 60˚C to 95˚C with a speed of 1.9 ˚C min^{-1}, resulting in melting curves. Data analysis was performed using SDS 2.4 software (Applied Biosystems Deutschland). Data were normalised on the basis of results obtained from measurements of standard material that was included in all performed experiments. The standard samples were collected from *Arabidopsis thaliana* plants, ecotype Col-0, grown on soil in a glasshouse.

d. Data calibration to absolute values

The transcript data are expressed in RNA copy number per cell. This absolute quantification of the transcripts level was achieved using artificial poly$(A)^+$ RNAs (ArrayControl *RNA Spikes* - Applied Biosystems) as internal standards. Eight different artificial mRNA spikes with known concentration (Supplementary Table 1) were mixed and the same amount of the spikes mix was added to all tested samples at the first step of the RNA extraction procedure (after adding the RLC buffer to the frozen plant material, see above). The efficiency and the C_t values of all 8 spikes were used to generate a standard curve for each sample. Each measured data point was plotted against the standard curve to calculate the concentration of mRNA in units of copy number per gram fresh weight (copy number / g FW). The procedure is very similar to our earlier report (2), which also addressed the accuracy of the measurements. Our results are in line with their conclusion that, for a single timepoint with achievable replication, "Precise quantification of changes of [less than] two-fold is difficult using qRT–PCR.". Adjacent timepoints and successive daily cycles in circadian data provide greater support than the single-timepoint assays in (2). Rigorous assessment of the uncertainties will require more sophisticated statistical models, as the system's behaviour is not independent at successive, 2-h timepoints.

To determine the cell number per g FW, the genome copy number per gFW was quantified. For this purpose artificial DNA spikes were added to the extract during DNA extraction (at the beginning of the protocol). Next, PCR reactions with primers for genes that are known to have only a single copy

per genome (e.g. mitochondrial 26S ribosomal RNA protein, NADH dehydrogenase subunit 9, NAD(P)H dehydrogenase subunit H protein) were performed. The ploidy level of the samples was estimated using flow cytometry. Finally, the genome copy number per g FW was divided by the ploidy level. The resulting number (25.000.000 copy number/g FW) was used to express metabolite and transcript data.

McWatters dataset (Experiment 5 of Figure 1C)

Seedlings were grown on sterile media containing 3% sucrose. Entire seedlings including roots were harvested every 3h. RNA was extracted, amplified and analysed, all using methods previously described (3).

Edwards dataset (Experiment 6 of Figure 1C)

Seedlings were grown on sterile media containing 3% sucrose. Entire seedlings including roots were harvested every 2h. RNA was extracted and analysed as described (4).

Southern dataset (Experiment 7 of Figure 1C)

Seedlings were grown on sterile media containing 3% sucrose, harvested every 3h with additional sampling times around light-dark transitions; RNA was extracted, amplified and analysed, as described (5).

Evaluation of datasets

The larger data sets reported here were acquired using automated, quantitative RT-PCR platforms that were established as shared facilities for research centres in Golm and Edinburgh. The experiments remained laborious and costly, despite the automation and reduced reagent costs from small assay volumes; further miniaturisation of these assays shows promise for future studies (as in 6). Large-scale data cannot usually be acquired with the same uniformity as small-scale datasets. Replacing individual measurements with supplementary experiments to improve technical quality is infeasible, because the broad scope of genotypes, conditions and timepoints in the original studies makes such experiments expensive relative to the small number of improved data points, and introduces a further source of variation. Thus we note technical variation in order to inform subsequent analysis, reinforcing the point that only a small proportion of the data were affected. RNAs with low amplitude regulation (*TOC1*, *ELF3*) or low expression levels (*PRR9*) are most obviously affected. Automated liquid handling avoided the sample-to-sample variation of manual pipetting in the earlier, Southern data (Supplementary Figure 5E, 5F) but risked introducing larger-scale effects. Three issues affected multiple samples. One timepoint (ZT24) in the ROBuST data was discarded for several genotypes due

to a harvesting error; ZT24 data are expected to be very similar to ZT0 but the tissue is one day older. Some technical replicates in ROBuST were discarded and repeated wholesale when a trend was discovered across multiple samples, due to a progressive liquid handling error in the robot used in PCR setup. The first Col-0 samples in the TiMet rosette dataset showed high biological variability due to harvesting or calibration, so the entire first 24h of the timeseries were replaced by a supplementary experiment (shown as experiment 2B in Figure 1C).

No molecular phenotype was detected when the *toc1* mutant was tested under red light in the Southern data set (data not shown). The strong *toc1-2* mis-sense allele that was used in this study causes a splicing defect (7). The expected change in the size of the *TOC1* transcript was detected using the method described (7) on our RNA samples, confirming their genotype (8). *toc1-2* seedlings do show hypocotyl elongation defects when grown under red light on media without exogenous sucrose (9), so the absence of molecular phenotype is possibly due to 3% sucrose in our media. The *toc1-9* and *toc1-100* alleles tested under white light in the ROBuST and TiMet data sets gave the expected molecular phenotypes.

We found no consistent effect on the RNA profiles from normalisation to endogenous control RNAs. TiMet rosette data were normalised to absolute, external standards (Figure 1) or to internal controls (Figure 3), for comparison to the profiles from other data sets. The profiles were very similar in both analyses, even for features that varied across experiments. In the GI profile, for example, the minor peak at ZT2 was 20% of the major peak at ZT8 in Figure 1 in Col in Figure 1 (18% in Figure 3), or 11% (10%) in Ws. The 1-2% differences were much less than the biological variation (see Figure 1).

Supplementary Analysis

Interpretation of Phase-Plane Diagrams

Phase plane diagrams plot the levels of two components against each other (Figure 7), exactly as in a scatter plot used to test for correlation. The data in scatter plots do not usually have a meaningful sequence. Timeseries do, so the data points in a phase plane diagram are connected by a line that represents their time sequence. Data symbols for ZT0 and ZT12 are enlarged to provide time references, because sample times are not directly plotted. Rhythmic profiles in phase lead to a diagonal plot with a positive gradient, for example for *GI* and *TOC1* expression (Figure 7A), whereas rhythms 90 or 270 degrees out of phase produce a circular plot. *GI* and *TOC1* expression reached peak and trough levels at overlapping timepoints, elongating the plot along the positive diagonal. The plots form an open shape rather than a line, because *GI* rose without (before) *TOC1*, especially in Col plants of the TiMet and ROBuST data sets, without exogenous sucrose. High *TOC1* occurred after

high *GI*, particularly in Ws plants of the TiMet data sets. This relative timing is of course easy to see in conventional timeseries plots, and has been noted in many past studies.

Rhythms 180 degrees out of phase yield a right-angled plot on a linear scale, or a diagonal of gradient -1 on a logarithmic scale, illustrated in the plots of *CCA1* and *TOC1* RNA (Figures 7B, 7C) The logarithmic scale shows that *CCA1* expression rises over 100-fold along roughly horizontal lines, indicating little effect on *TOC1*. In contrast, the rise in *TOC1* expression directly correlates with falling *CCA1,* resulting in diagonals with negative gradients (Figure 7C). Linear scale plots might best represent potential protein synthesis, which is usually assumed to increase linearly with mRNA level; hence linear plots suggest likely effects on downstream targets. On the other hand, logarithmic scale plots reveal the effects of upstream regulators, even at low RNA levels that are indistinguishable on a linear scale.

The dependence of an interaction of two genes upon a third regulator was strikingly illustrated by plotting the profiles of *PRR9* and *ELF4,* which peak far out of phase in the wild type (Figure 7D). Starting from ZT0, acute light activation of *PRR9* drives peak expression at ZT2-6 while *ELF4* expression is minimal. *ELF4* expression rises to peak at ZT10 as *PRR9* falls, consistent with some contribution to repressing *PRR9*. *ELF4* expression falls at night (red dashed line) while *PRR9* expression is minimal. Data from LL (filled symbols) suggest a negative correlation in the subjective night, when *ELF4* falls as *PRR9* rises. In the *lhy cca1* double mutant under LD cycles (Figure 7E, open symbols), *PRR9* is partially de-repressed at ZT0, but remains out of phase with *ELF4.* However, the two genes are co-regulated for part of the cycle under LL, creating a diagonal with a positive gradient (red dashed line, Figure 6F).

Phase plane diagrams for the *prr7 prr9* double mutant (Figure S6) showed that *ELF4* expression rose while *CCA1* (and *LHY,* not shown) were still significantly expressed at the end of the day, suggesting that not only CCA1 and LHY but also the PRRs repress *ELF4* in the wild type. Removing *PRR9* and *PRR7* also revealed simpler correlations of *PRR5* expression with *CCA1* and *ELF4* (Figures S6E, S6H).

Model versioning and optimisation

We have adapted the Community Earth System Model version-naming convention (10) (www2.cesm.ucar.edu/models) to describe version of our clock models. Models are named as Axxxx.Y.Z., where A is a letter of the model author’s surname, xxxx is the year of submission, Y is a version number marking a significant change in the model that alters its dynamics such as a new

parameter set, Z is a minor change that does not alter the model's dynamics such as the refactoring of the light input function. The version identifier should link to a unique identifier in at least one model repository.

Model optimisation used SBSIvisual version 1.4.5 (11) and SBSInumerics version 1.2, which are both available from Sourceforge. Model P2011.1.2 (PLM_64, version 4) was optimised using SBSI's Parallel Genetic Algorithm, with a range of parameter constraints (Table 2). Starting parameter sets were chosen as normal (P2011.2.1) or logarithmic values (P2011.3-P2011.6) within the stated ranges, either randomly (P2011.3-P2011.6) or from a quasi-random Sobol series (P2011.2.1). The departure of each model's simulations of wild-type and mutants from the normalised TiMet rosette data was evaluated using SBSI's chi-square cost function, and their departure from measured period values under constant light was evaluated using SBSI's FFT cost function. SBSInumerics v1.2 estimates the period of simulated timeseries with greater precision than v1.1. SBSInumerics does not specifically cost the amplitude of the simulated timeseries, which are constrained only by the match to the RNA data. The results (Figure 9) suggest that small discrepancies in simulated amplitude are better tolerated in the costing than discrepancies in timing, matching our priorities. Moreover, our choice of Hill coefficients is very conservative (all set at 2), restricting the nonlinearity of simulated gene regulation and tending to reduce the amplitudes of the simulated RNA profiles. The >100-fold relative amplitudes observed for some clock RNAs suggest much greater non-linearity of regulation in vivo.

Optimisation job .599 tested 5000 randomly-generated parameter sets and optimised the best solutions in a population of 200 models over 599 generations; computation time was 6 hours on a server with eight Intel Xeon processing cores. Job t40 tested randomly-generated parameter sets before optimising the best solutions as above; computation time was 8.5 hours using 4096 processing cores on the UK national supercomputing resource HECToR. The parameter constraints and optimised parameter values are listed in the SBML files (see Data, model and code accessibility).

Supplementary Figure Legends

Supplementary Figure 1. Overview of the clock gene circuit

The clock gene circuit summarised in the Activity-Flow language of SBGN v1.0 (12), with the principal light inputs and regulatory interactions in the P2012 model (13). The repressilator is denoted by green lines; morning loop components are filled yellow; evening loop components are filled blue.

Supplementary Figure 2. Detailed schema of the P2011 model

The clock gene network in the P2011 model (14), represented in the SBGN Process Description language. All connections in the model are depicted, along with the logic inherent in the form of the equations. Coloured edges correspond to different parts of the model: Yellow edges - Light inputs. Green edges - Morning loop. Blue edges - Evening loop. Purple edges – connections among loops.

Supplementary Figure 3. Logarithmic plots of clock gene expression in wild-type plants under LD cycles

Transcript levels in Col-0 and Ws-2 wild types under LD 12:12 were measured by qRT-PCR, in experiment 2 (TiMet ros) including eight external RNA standards to allow absolute quantification in Col-0 and Ws-2 (A, C, E) and in experiment 1 (ROBuST) normalised to the *ACTIN7* control in Col-4 and Ws-2 (B, D, F). Data represent transcripts of (A, B) *LHY* and *CCA1*, (C, D) *PRR9*, (E, F) *TOC1* and *GI*. Error bars show SD, for 2-3 biological replicates. Figure 2 shows the same data on a linear scale.

Supplementary Figure 4. RNA profiles under DD

RNA profiles of (A) *CCA1* and (B) *GI* expression from the Edwards dataset for seedlings on sucrose (4), under LD cycles of 6, 12 or 18h photoperiod followed by DD (legend, panel B). RNA profiles from the TiMet rosette dataset for plants in 12h photoperiod, transferred to DD, for (C) *PRR7*, (D) *PRR9* expression. Higher trough levels are observed in DD than under LD, often more than ten-fold higher. (A), (B) Data shown are relative to the *ACTIN2* control; (C)-(D) data are calibrated to RNA copy number per cell. Means of two biological replicates per timepoint are shown, error bar is SD. Open box, light interval; filled box, dark interval, A and B, coloured as for legend.

Supplementary Figure 5. Mutant effects are consistent among data sets.

RNA profiles from the ROBuST dataset for seedlings under LD cycles show (A) the slower fall of *PRR5* expression in the *gi-11* mutant compared to wild type, persisting from ZT10-16h (logarithmic scale, B); (C) earlier and lower peak expression of *TOC1* in the *lhy cca1* double mutant; (D) lower expression of *ELF3* in the *gi* mutant, especially at ZT8-10h; (E) persistent expression of *CCA1* in the prr7 prr9 double mutant, with an early rise in *prr7* and delayed fall in *prr9*. RNA profiles from the Southern data for *elf3* and *elf4* mutant seedlings under red light-dark cycles show (F) persistently high and noisy expression of *GI*; (G) expression of *CCA1* that falls from the level in Ws

to *elf4*, which remains rhythmic in constant conditions, and falls further to *elf3*, which is arrhythmic. A-D show means of three biological replicates per timepoint, error bars are SEM. E, F show one of two replicate experiments with similar results. Plots in panels C and D are from BioDare.

Supplementary Figure 6. Phase plane diagrams reveal altered regulation in *prr7 prr9* mutants.

RNA profiles of Figure 5 are represented as phase plane diagrams on logarithmic scales, plotting data for *ELF4* and *CCA1* (A) in wild-type Col plants under LD and LL and (B) in Col plants under LD and *prr7 prr9* double mutants under LD and LL, with (C) the LD data for both genotypes on linear scales.

(D)-(E) show data for *PRR5* and *CCA1* (D) in wild-type Col plants under LD and LL and (E) in Col plants under LD and *prr7 prr9* double mutants under LD and LL. (E) Red dashed line marks anti-correlated levels during the subjective night in the double mutant in LL.

(F)-(H) show data for *ELF4* and *PRR5* (F) in wild-type Col plants under LD and LL, (G) in Col plants under LD and *prr7 prr9* double mutants under LD and (H) for *prr7 prr9* double mutants under LD and LL. (H) Red dashed lines mark highly correlated rise and fall of *PRR5* and *ELF4* levels in the double mutant under LL, whereas the relationship was more complex in the wild type. Larger markers indicate ZT0(24) and ZT12(36) datapoints in LD (LL), arrows indicate the direction of time.

Supplementary Figure 7. Regulation of clock-related genes in low-sugar conditions.

RNA microarray data (15, 16) displayed by the online tool (http://mapman.mpimp-golm.mpg.de/supplement/xn/) from treatments with light, CO_2-free air (ΔCO_2), DD (eXtended Night), or the starchless *pgm* mutant, for (A) *LHY*, *PRR7* (green), *PRR5* and *TOC1*; (B) *ZTL, FKF1* and *LKP2*. (A) Arrows mark higher *PRR7* levels in sugar-limiting DD and *pgm* relative to control in LD cycle, repression by re-supply of high exogenous sugar (3 Suc) but less effect from resupply of normal air (350ppm CO_2). (B) Arrows mark higher levels of *ZTL*, *FKF1* and *LKP2* RNA in DD and *pgm.*

Supplementary Table Legends

Supplementary Table 1. mRNA calibration standards.

Concentration of artificial mRNA spikes (number of copies per extract) added to each sample as an internal standard to allow the quantification of RNA copy number.

Supplementary Table 2. qRT-PCR primer sequences.

The PCR primer sequences used in the TiMet studies are listed below. The PCR primer sequences used in the ROBuST data set are listed in the public record for each transcript on BioDare (please see Data Accessibility), and the primer sequences for other studies are listed in their original publications and in the BioDare records (please see Data Accessibility).

Supplementary References

1. Dixon LE, Knox K, Kozma-Bognar L, Southern MM, Pokhilko A, Millar AJ. Temporal repression of core circadian genes is mediated through EARLY FLOWERING 3 in Arabidopsis. Curr Biol. 2011;21(2):120-5.
2. Piques M, Schulze WX, Hohne M, Usadel B, Gibon Y, Rohwer J, et al. Ribosome and transcript copy numbers, polysome occupancy and enzyme dynamics in Arabidopsis. Mol Syst Biol. 2009;5:314.
3. Knight H, Thomson AJ, McWatters HG. Sensitive to freezing6 integrates cellular and environmental inputs to the plant circadian clock. Plant Physiol. 2008;148(1):293-303.
4. Edwards KD, Akman OE, Knox K, Lumsden PJ, Thomson AW, Brown PE, et al. Quantitative analysis of regulatory flexibility under changing environmental conditions. Mol Syst Biol. 2010;6:424.
5. Locke JC, Southern MM, Kozma-Bognar L, Hibberd V, Brown PE, Turner MS, et al. Extension of a genetic network model by iterative experimentation and mathematical analysis. Mol Syst Biol. 2005;1:2005 0013.
6. Huang W, Perez-Garcia P, Pokhilko A, Millar AJ, Antoshechkin I, Riechmann JL, et al. Mapping the core of the Arabidopsis circadian clock defines the network structure of the oscillator. Science. 2012;336(6077):75-9.
7. Strayer C, Oyama T, Schultz TF, Raman R, Somers DE, Mas P, et al. Cloning of the *Arabidopsis* clock gene *TOC1*, an autoregulatory response regulator homolog. Science. 2000;289(5480):768-71.
8. Southern MM. Mutants in the Arabidopsis Circadian Clock: Genetic Approaches to Explore Circadian Mechanisms in the Model Higher Plant: University of Warwick; 2005; Ph.D. thesis.
9. Ito S, Nakamichi N, Nakamura Y, Niwa Y, Kato T, Murakami M, et al. Genetic linkages between circadian clock-associated components and phytochrome-dependent red light signal transduction in Arabidopsis thaliana. Plant Cell Physiol. 2007;48(7):971-83.
10. Levis S, Bonan GB, Kluzek E, Thornton PE, Jones A, Sacks WJ, et al. Interactive Crop Management in the Community Earth System Model (CESM1): Seasonal Influences on Land-Atmosphere Fluxes. J Climate. 2012;25(14):4839-59.
11. Adams R, Clark A, Yamaguchi A, Hanlon N, Tsorman N, Ali S, et al. SBSI: an extensible distributed software infrastructure for parameter estimation in systems biology. Bioinformatics. 2013;29(5):664-5.
12. Le Novere N, Hucka M, Mi H, Moodie S, Schreiber F, Sorokin A, et al. The Systems Biology Graphical Notation. Nat Biotechnol. 2009;27(8):735-41.
13. Pokhilko A, Mas P, Millar AJ. Modelling the widespread effects of TOC1 signalling on the plant circadian clock and its outputs. BMC Syst Biol. 2013;7:23.
14. Pokhilko A, Fernandez AP, Edwards KD, Southern MM, Halliday KJ, Millar AJ. The clock gene circuit in Arabidopsis includes a repressilator with additional feedback loops. Mol Syst Biol. 2012;8:574.
15. Blasing OE, Gibon Y, Gunther M, Hohne M, Morcuende R, Osuna D, et al. Sugars and circadian regulation make major contributions to the global regulation of diurnal gene expression in Arabidopsis. Plant Cell. 2005;17(12):3257-81.
16. Usadel B, Blasing OE, Gibon Y, Retzlaff K, Hohne M, Gunther M, et al. Global Transcript Levels Respond to Small Changes of the Carbon Status during Progressive Exhaustion of Carbohydrates in Arabidopsis Rosettes. Plant Physiol. 2008;146(4):1834-61.

Supplementary Tables

Supplementary Table 1

Concentration of artificial mRNA spikes (number of copies per extract) added to each sample as an internal standard to allow the quantification of RNA copy number.

	**Spike1**	**Spike 2**	**Spike 3**	**Spike 4**	**Spike 5**	**Spike 6**	**Spike 7**	**Spike 8**
**Copies per extract**	6.08 E+09	1.52 E+09	4.56 E+08	1.14 E+08	2.94 E+07	3.04 E+05	2.58 E+06	7.60 E+05

Supplementary Table 2. qRT-PCR primer sequences.

The PCR primer sequences used in the TiMet studies are listed below, followed by the PCR primers for the Southern dataset. The PCR primer sequences used in the ROBuST data set are listed in the public record for each transcript on BioDare (please see Data Accessibility), and the primer sequences for other studies are listed in their original publications and/or in the BioDare records.

Primers for the TiMet datasets.

Product	CODE	SEQUENCE	Comments
Spike 1	H251-A1780s1-f1	GATGCCCGACCATCATTTAG	
	H252-A1780s1-r1	GTGAGGGTAATGTCGCGTTC	
Spike 2	H253-A1780s2-f1	ATTGCGCTCGCCATATACAC	
	H254-A1780s2-r1	GCTGGGATCAGGAGGAGAAG	
Spike 3	H255-A1780s3-f1	GATCGTTTGCCTGCATTACC	
	H256-A1780s3-r1	GAGAGCGTCAGCCATACCAC	
Spike 4	H257-A1780s4-f1	TACGTCGCACAACCACAATC	
	H258-A1780s4-r1	CAGCGCCACTAACCCACTAC	
Spike 5	H302-S5f-2	ACAAAGGCAGCGTTGAAAAC	
	H301-S5r-1	TAGTGTCTGCACGCCATACC	
Spike 6	Spike-6f-2	CGCAAAGTCTCTCCTCTTGG	
	Spike-6r-2	CAGTAGCCATTGCGGAAGAT	
Spike 7	H313-S7f-3	CTGAACCAGACTGCACATGG	
	H314-S7r-3	ACGCTCATGGGCTTGTTTAT	
Spike 8	H315-S8f-3	TCATTAAAGCGGAAGGCAAT	
	H316-S8r-3	AAATCTTCCAGCACCGTCAG	
GAPDH 3'	H278-GAPDH3	TTGGTGACAACAGGTCAAGCA	
	H279-GAPDH3	AAACTTGTCGCTCAATGCAATC	
GAPDH 5'	H280-GAPDH5	TCTCGATCTCAATTTCGCAAAA	
	H281-GAPDH5	CGAAACCGTTGATTCCGATTC	
ACT2 155	H282-155-ACT2	AACTCTCCCGCTATGTATGTCGC	
	H283-155-ACT2	CAATACCGGTTGTACGACCACTG	
ACT2 633	H284-633-ACT2	ACTTTCATCAGCCGTTTTGA	

	H285-633-ACT2	ACGATTGGTTGAATATCATCAG	
LHY	F019-LHY-f	TGCCTCAAAGCTTTTCGCCTCCT	
	F020-LHY-r	GTCTGCAGCACAAGAATCCTGGCT	
CCA1	F027-CCA1-f	TCCAATGCACGCCGCAGTAGAA	
	F028-CCA1-r	AGGCAATTCGACCCTCGTCAGACA	
PRR9	F037-PRR9-f	AGCTAGCAGAACAACGTCCTCGAGT	
	F038-PRR9-r	CGTCTGAATTCACGGTTCGCACGA	
PRR9	JF309-PRR9-F	GATTGGTGGAATTGACAAGC	primers from A. Millar
	JF310-PRR9-R	TCCTCAAATCTTGAGAAGGC	
PRR7	F045-PRR7-f	AGAGGTGCTTCCGAAAGAAGGTACGA	
	F046 -PRR7-r	ACGCACAAATTGGCCTCGCACT	
PRR5	F051-PRR5-f	TGCAATGGCTCCTGCTTCACTCTC	
	F052-PRR5-r	ACTGTACTCATGCGGGCTAACGGA	
TOC1	F073-TOC1-f	TGATGGATCGGGTTTCTCTGCACCA	
	F074-TOC1-r	TGAGGCATCATGGCTGCTGATTGC	
LUX	F077-LUX-f	CAGCGGTAATGTTGGAGTGCCGAT	
	F078-LUX-r	TGGCATCTGCATCATCTGTTGCGT	
GI	F083-GI-f	TGCGGGCAACTGATGGAATGCT	
	F084-GI-r	TGCTCTTGCCGTGGCTTCAAGT	
ELF4	F091-ELF4-f	AGTTTCTCGTCGGGCTTTCACGGT	
	F092-ELF4-r	TAAGCTCTAGTTCCGGCAGCACCA	
ELF3	F099-ELF3-f	ACAACAAGAGATGGGGGAGGAGTGAC	
	F100-ELF3-r	ACTCGCGAGCTTTGCGTTGTGA	
ELF3	JF576-ELF3-F	CATTCGCAGCCGTTGATGAGG	from Coluccio et al. J. Exp.Bot, 2011
	JF577-ELF3-R	TGTTCTTGTCGTCGTTGTGGTTG	

Primers for the Southern dataset (8).

Product	Forward primer	Reverse primer
ACT2	CAGTGTCTGGATCGGAGGAT	TGAACAATCGATGGACCTGA
LHY	CAACAGCAACAACAATGCAACTAC	AGAGAGCCTGAAACGCTATACGA
CCA1	CTGTGTCTGACGAGGGTCGAA	ATATGTAAAACTTTGCGGCAATACCT
GI	AATTCAGCACGCGCCTATTG	GTTGCTTCTGCTGCAGGAACTT
TOC1	ATCTTCGCAGAATCCCTGTGATA	GCACCTAGCTTCAAGCACTTTACA
ELF3	GGAAAGCCATTGCCAATCAA	ATCCGGTGATGCAGCAATAAGT
ELF4	CGACAATCACCAATCGAGAATG	AATGTTTCCGTTGAGTTCTTGAATC

Supplementary Figure 1

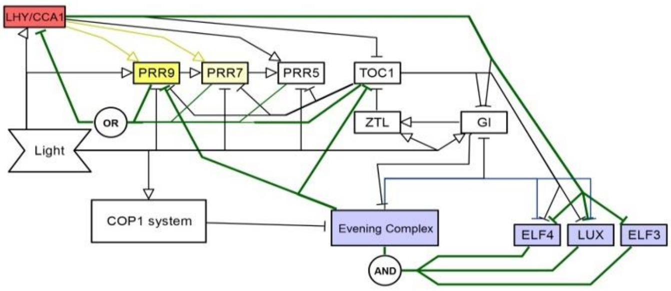

Supplementary Figure 1. Overview of the clock gene circuit

The clock gene circuit summarised in the Activity-Flow language of SBGN v1.0 (Le Novere *et al.*, 2009), with the principal light inputs and regulatory interactions in the P2012 model (Pokhilko *et al.*, 2013). The repressilator is denoted by green lines; morning loop components are filled yellow; evening loop components are filled blue.

Supplementary Figure 2

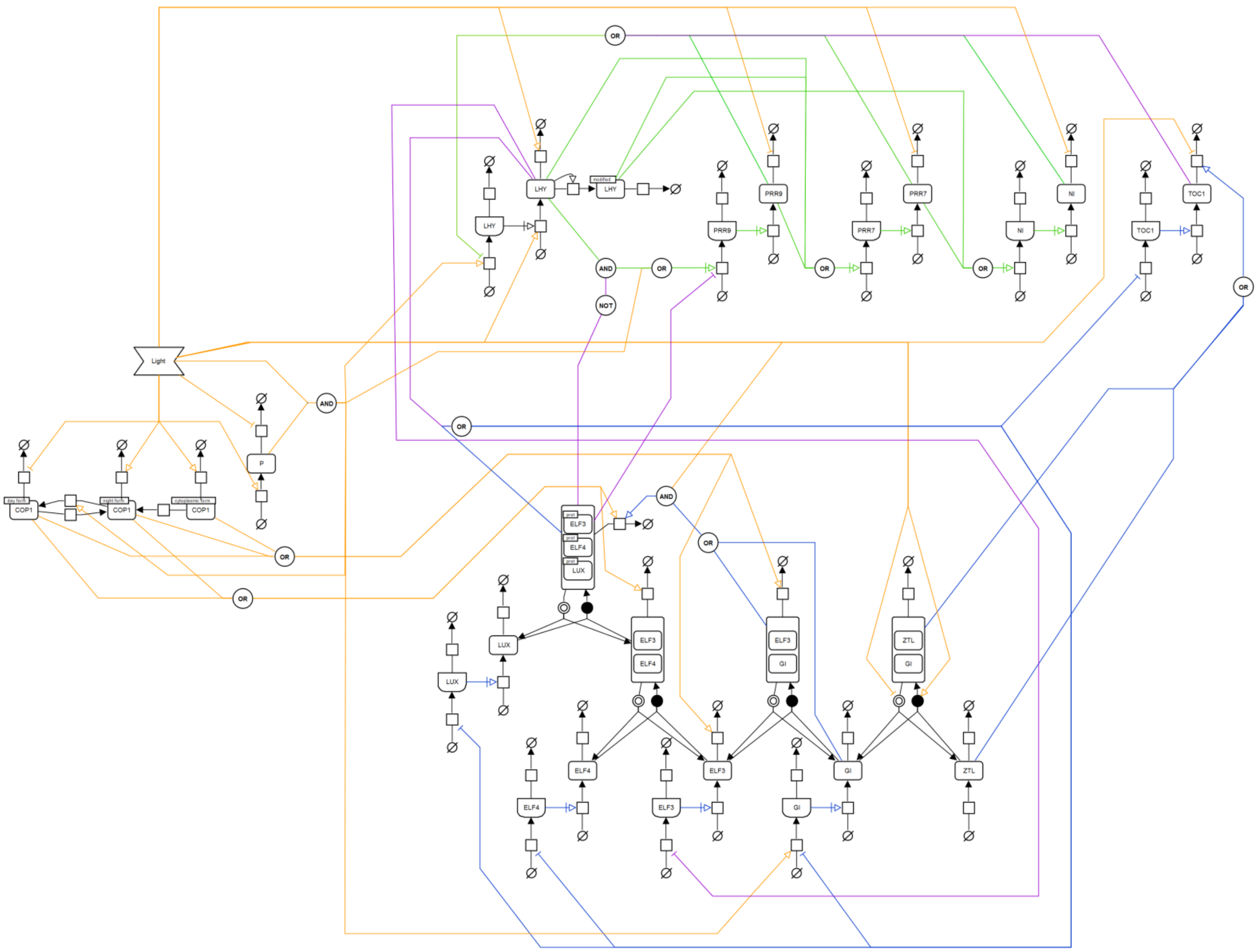

Supplementary Figure 2. Detailed schema of the P2011 model

The clock gene network in the P2011 model (Pokhilko *et al.*, 2012), represented in the SBGN Process Description language. All connections in the model are depicted, along with the logic inherent in the form of the equations. Coloured edges correspond to different parts of the model: Yellow edges - Light inputs. Green edges - Morning loop. Blue edges - Evening loop. Purple edges – connections among loops.

Supplementary Figure 3

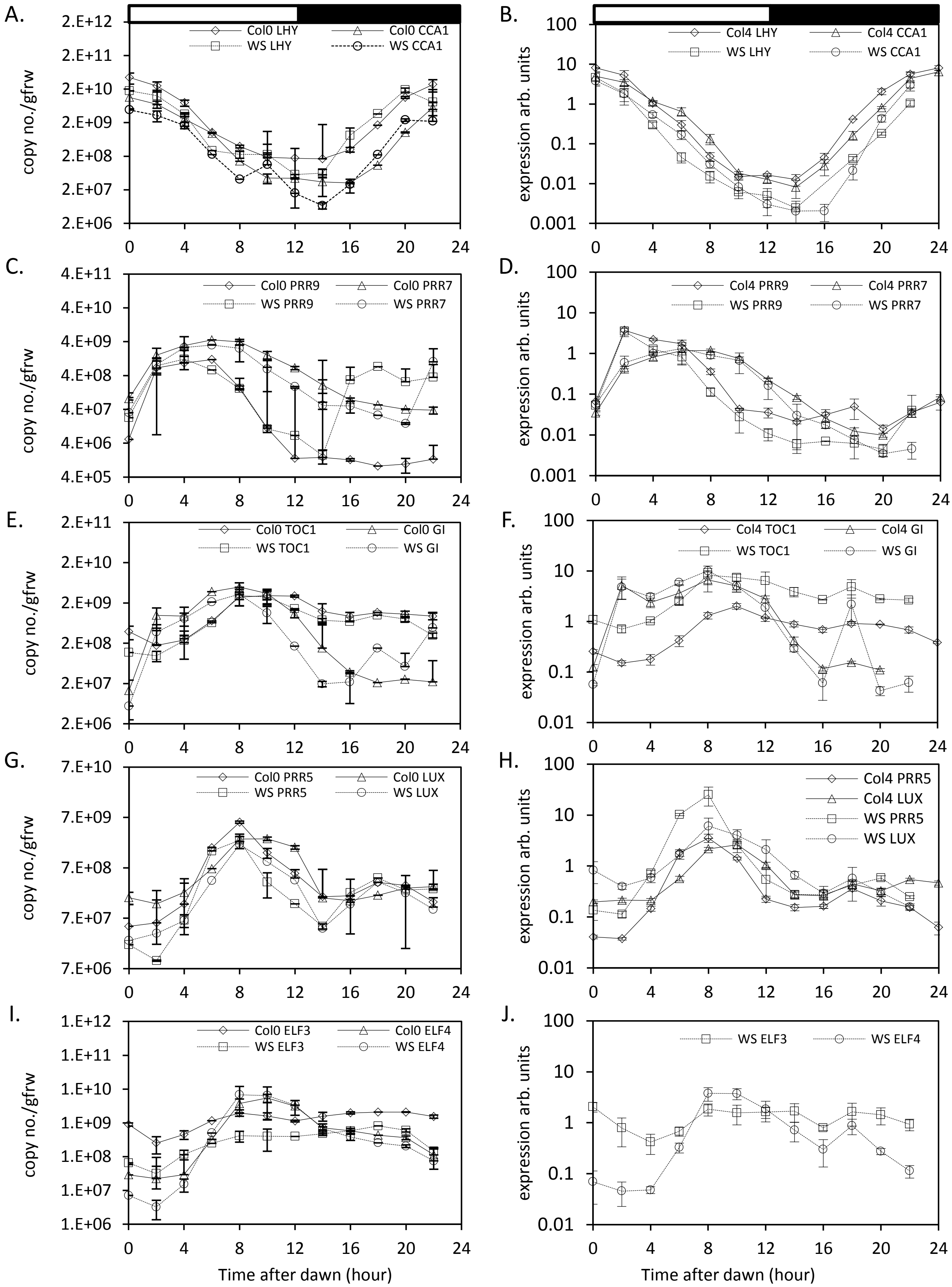

Supplementary Figure 3. Logarithmic plots of clock gene expression in wild-type plants under LD cycles
Transcript levels in Col-0 and Ws-2 wild types under LD 12:12 were measured by qRT-PCR, in experiment 2 (TiMet ros) including eight external RNA standards to allow absolute quantification in Col-0 and Ws-2 (A, C, E) and in experiment 1 (ROBuST) normalised to the *ACTIN7* control in Col-4 and Ws-2 (B, D, F). Data represent transcripts of (A, B) *LHY* and *CCA1*, (C, D) *PRR9*, (E, F) *TOC1* and *GI*. Error bars show SD, for 2-3 biological replicates. Figure 2 shows the same data on a linear scale.

Supplemental Figure 4

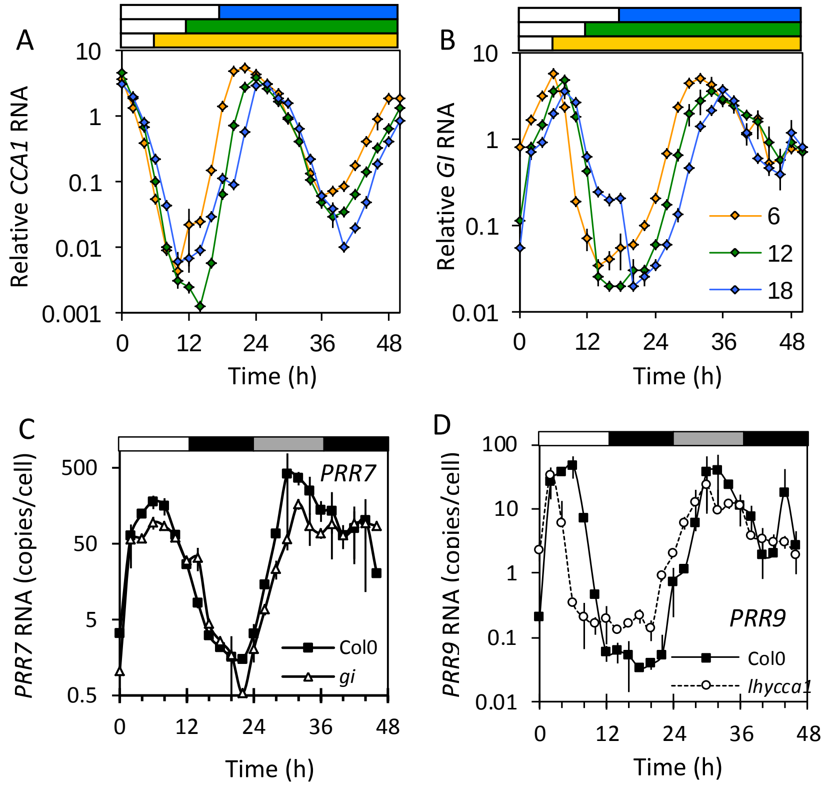

Supplementary Figure 4. RNA profiles under DD

RNA profiles of (A) *CCA1* and (B) *GI* expression from the Edwards dataset for seedlings on sucrose (Edwards *et al.*, 2010) under LD cycles of 6, 12 or 18h photoperiod followed by DD (legend, panel B). RNA profiles from the TiMet rosette dataset for plants in 12h photoperiod, transferred to DD, for (C) *PRR7,* (D) *PRR9* expression. Higher trough levels are observed in DD than under LD, often more than ten-fold higher. (A), (B) Data shown are relative to the *ACTIN2* control; (C)-(D) data are calibrated to RNA copy number per cell. Means of two biological replicates per timepoint are shown, error bar is range. Open box, light interval; filled box, dark interval, A and B, coloured as for legend.

Supplementary Figure 5

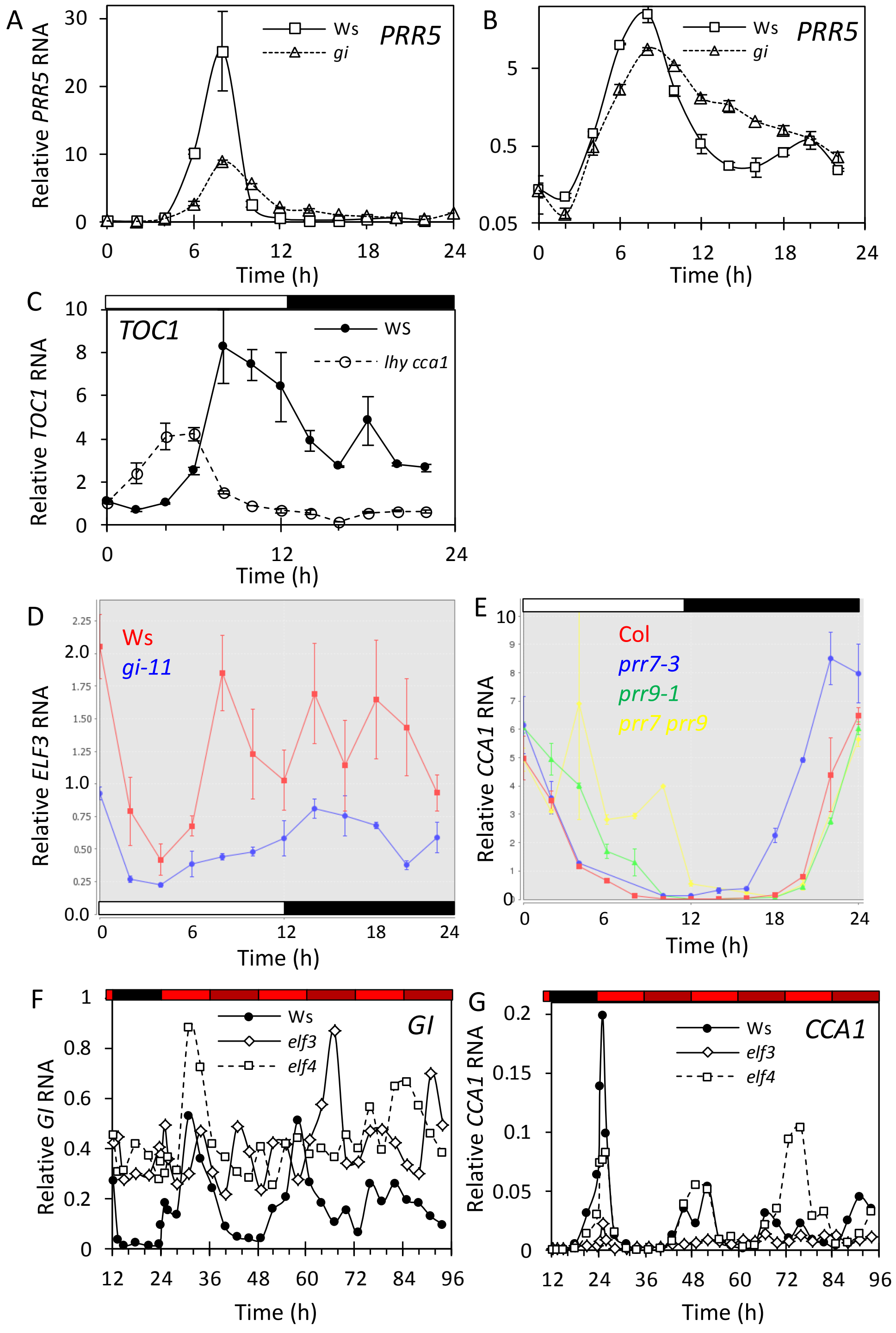

Supplementary Figure 5. Mutant effects are consistent among data sets.
RNA profiles from the ROBuST dataset for seedlings under LD cycles show (A) the slower fall of *PRR5* expression in the *gi-11* mutant compared to wild type, persisting from ZT10-16h (logarithmic scale, B); (C) earlier and lower peak expression of *TOC1* in the *lhy cca1* double mutant; (D) lower expression of *ELF3* in the *gi* mutant, especially at ZT8-10h; (E) persistent expression of *CCA1* in the prr7 prr9 double mutant, with an early rise in *prr7* and delayed fall in *prr9*. RNA profiles from the Southern data for *elf3* and *elf4* mutant seedlings under red light-dark cycles show (F) persistently high and noisy expression of *GI*; (G) expression of *CCA1* that falls from the level in Ws to *elf4*, which remains rhythmic in constant conditions, and falls further to *elf3*, which is arrhythmic. A-D show means of three biological replicates per timepoint, error bars are SEM. E, F show one of two replicate experiments with similar results. Plots in panels C and D are from BioDare.

Supplementary Figure 6

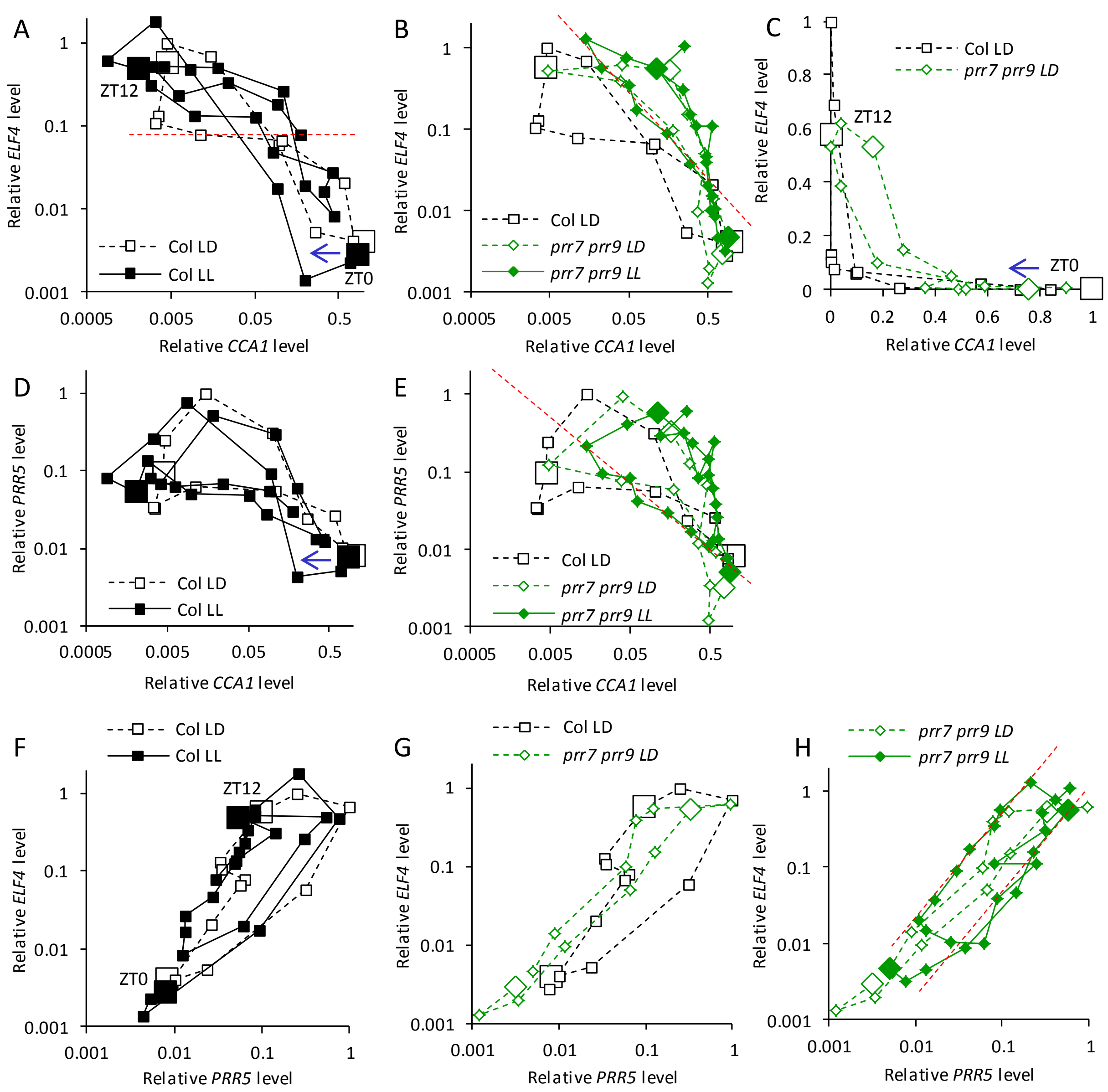

Supplementary Figure 6. Phase plane diagrams reveal altered regulation in *prr7 prr9* mutants.
RNA profiles of Figure 5 are represented as phase plane diagrams on logarithmic scales, plotting data for *ELF4* and *CCA1* (A) in wild-type Col plants under LD and LL and (B) in Col plants under LD and *prr7 prr9* double mutants under LD and LL, with (C) the LD data for both genotypes on linear scales.
(D)-(E) show data for *PRR5* and *CCA1* (D) in wild-type Col plants under LD and LL and (E) in Col plants under LD and *prr7 prr9* double mutants under LD and LL. (E) Red dashed line marks anti-correlated levels during the subjective night in the double mutant in LL.
(F)-(H) show data for *ELF4* and *PRR5* (F) in wild-type Col plants under LD and LL, (G) in Col plants under LD and *prr7 prr9* double mutants under LD and (H) for *prr7 prr9* double mutants under LD and LL. (H) Red dashed lines mark highly correlated rise and fall of *PRR5* and *ELF4* levels in the double mutant under LL, whereas the relationship was more complex in the wild type. Larger markers indicate ZT0(24) and ZT12(36) datapoints in LD (LL), arrows indicate the direction of time.

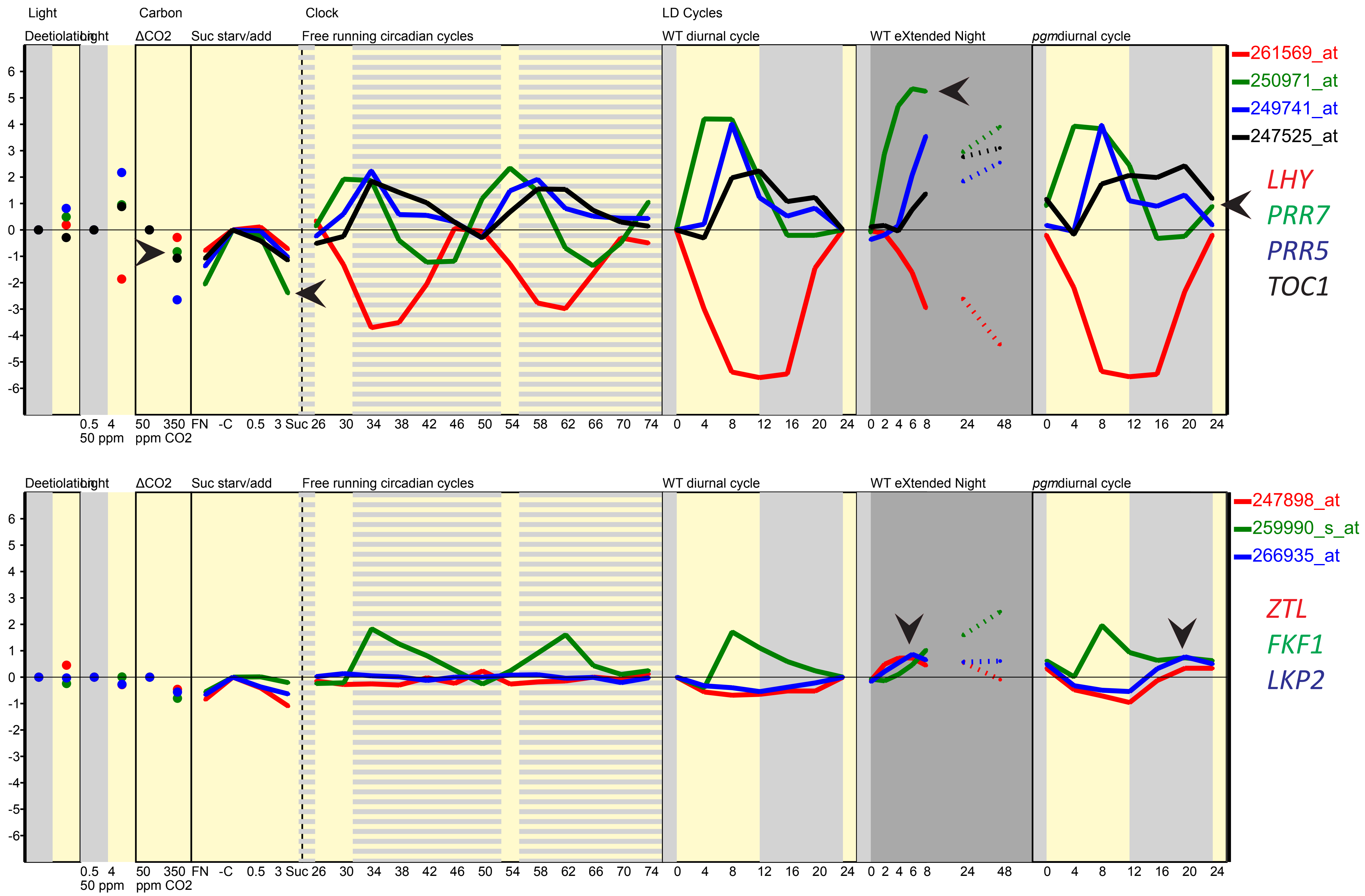

Light
Carbon
Clock
LD Cycles
Deetiolation
Light
ΔCO2
Suc starv/add
Free running circadian cycles
WT diurnal cycle
WT eXtended Night
pgm diurnal cycle
261569_at
250971_at
249741_at
247525_at
LHY
PRR7
PRR5
TOC1
0.5 4
50 ppm
50 350
ppm CO2
FN -C 0.5 3 Suc
26 30 34 38 42 46 50 54 58 62 66 70 74
0 4 8 12 16 20 24
0 2 4 6 8 24 48
0 4 8 12 16 20 24
247898_at
259990_s_at
266935_at
ZTL
FKF1
LKP2

Supplementary Figure 7. Regulation of clock-related genes in low-sugar conditions.
RNA microarray data (Blasing *et al.*, 2005; Usadel *et al.*, 2008) displayed by the online tool (http://mapman.mpimp-golm.mpg.de/supplement/xn/) from treatments with light, CO_2-free air (ΔCO_2), DD (eXtended Night), or the starchless *pgm* mutant, for (A) *LHY*, *PRR7* (green), *PRR5* and *TOC1*; (B) *ZTL*, *FKF1* and *LKP2*. (A) Arrows mark higher *PRR7* levels in sugar-limiting DD and *pgm* relative to control in LD cycle, repression by re-supply of high exogenous sugar (3 Suc) but less effect from resupply of normal air (350ppm CO_2). (B) Arrows mark higher levels of *ZTL, FKF1* and *LKP2* RNA in DD and *pgm.*
